# Supplementary material for: In vitro model reveals structural and metabolic insights into the porcine cecal microbiota in response to β-mannan exposure
Source: Appl Environ Microbiol. 2026 Jun 18;92(7):e00140-26. doi: 10.1128/aem.00140-26 (PMC13390407; doi:10.1128/aem.00140-26)
Supplement: Supplemental File A — In-depth analysis results. [file aem.00140-26-s0001.docx]

Supplementary file A

***In vitro* model reveals structural and metabolic insights to the porcine caecal microbiota in response to β-mannan exposure**

This supplementary file contains in-depth analysis results and explanations substantiating the claims made in the main text of the manuscript.

**Benchmarking: InVitSim did not introduce taxonomic nor functional biases to the *in vitro* microbial community.**

Alpha diversity measurements of the pre- and post-fermentation microbial community (**Fig. 2A**) showed that after 9 hours of fermentation in InVitSim, samples retained around 70% of the number of microbial species detected in the samples pre-fermentation. Conversely, the observed taxonomic and functional diversities had increased during the fermentation. To investigate these observations, we first assessed the microbial community compositions in InVitSim. Among the 301 taxa in the metagenome-assembled genome (MAG) catalogue, there were 242 types of Bacillota. The second most abundant phylum was Bacteroidota with 36 representatives. The Bacillota representatives and samples overall were dominated by the genera *Blautia*, *Eubacterium*, and *Agathobacter*; each counting 15 populations observed at around 5% relative abundance in all samples (**Fig. SA1**). However, the most numerous and abundant genus across the samples was the Bacteroidota genus *Prevotella*, represented by 29 distinct populations and a relative abundance of 10% or more in all samples. The strong prevalence of *Prevotella* is in accordance with previous studies of porcine gut microbiota ^[[1]](#footnote-2)^.


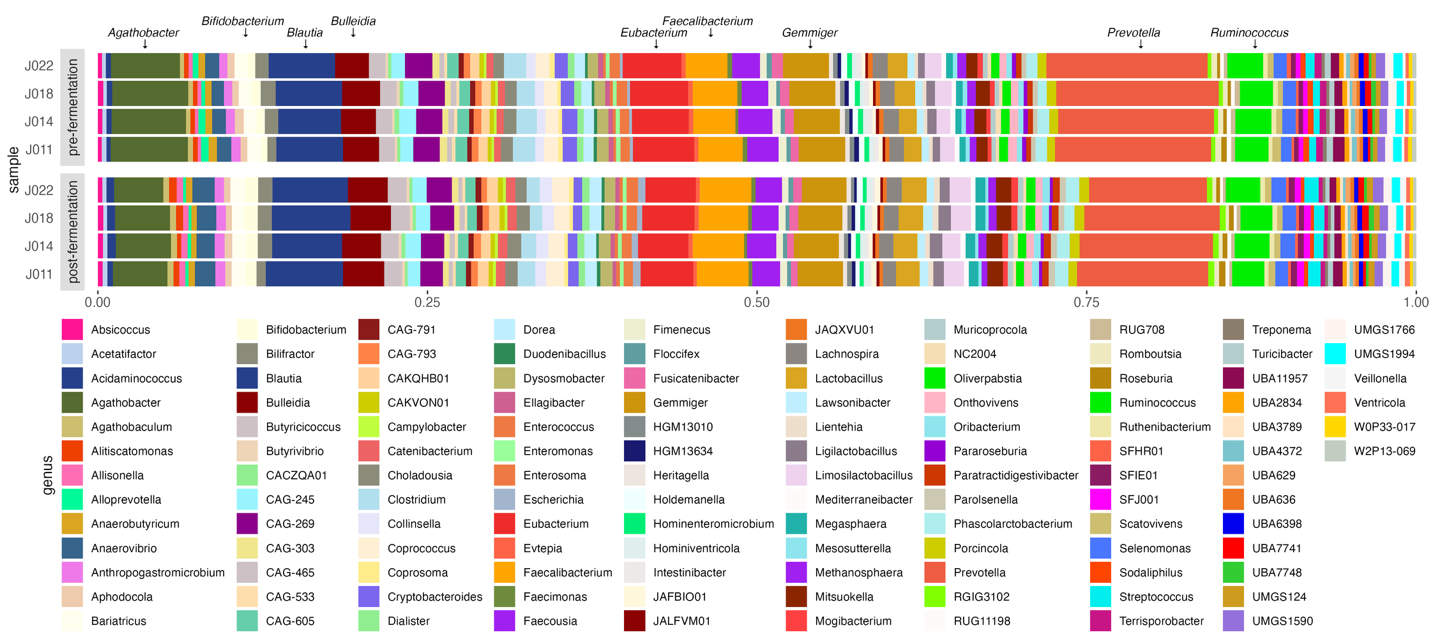


**Figure SA1**. *Relative abundances of genera in samples from pigs given a negative control diet, grouped according to whether the sample was extracted before or after the 9-hour in vitro fermentation. Each bar in the “post-fermentation” category comprises samples grown in control and AcGGM-supplemented media, leaving the sampling time point the central contrast in this comparison. Identical to Fig. 2B from the main text, but with the colour legend included.*

To quantify these relative abundace changes for taxa that appeared impacted by the *in vitro* setup, we performed a differential abundance analysis comparing pre- and post-fermentation samples from pigs given the negative control diet. The analysis revealed that 58 populations increased significantly in abundance, whereas 72 population sizes decreased significantly. The four most numerous and abundant genera in the initial samples were *Agathobacter, Blautia, Eubacterium*, and *Prevotella* (**Fig. SA1**). Together, they represented 10 and 32 of the populations with increased and decreased abundance, respectively; and all but *Blautia* had more populations with decreased than increased abundance. Assuming that the differential abundance between pre- and post-fermentation states reflects how well representatives of each genus persisted in InVitSim, the observation that more MAG abundances decrease over time aligns with the observed loss in species richness and Shannon entropy (**Fig. 2A**). This negative trend being strong for three of the four most dominating genera in turn contributed to increasing phylogenetic diversity (**Fig. 2A**), as the microbial community post-fermentation consisted of a more even population composition. Furthermore, the functional potential was tightly connected to taxonomy (**Fig. 2C**); hence the reduction of dominant genera also counteracted the enrichment of these taxa’s functional capabilities. In conclusion, the reduced abundance of some of the micorbial community’s most prominent members functioned as a balancing act that increased the relative presence of other community members, and contributed to a greater variation in taxa and functionality. The 70% of microbial taxa that persisted in the *in vitro* model represented a similar taxonomic and functional community to the pre-fermentation state. Hence the InVitSim approach did not introduce biases in the caecal microbiota.

**Benchmarking: Comparison of alpha diversity metrics across *in vivo* and *in vitro* microbial communities exposed to β-mannan.**

Using metagenomic data and the accompanying phylogenetic tree from Michalak *et al.* ^[[2]](#footnote-3)^, we calculated alpha diversities (**Fig. SA2**) to compare the effect of acetylated galactoglucomannan (AcGGM) supplements on the *in vivo* microbial gut community to observations from our InVitSim gut model (**Fig. 2A**). Both systems showed a reduced species richness and Shannon entropy from negative controls to AcGGM-supplemented samples. While the microbial community in InVitSim also displayed a lower phylogenetic diversity following AcGGM administration, this trend was not evident in the *in vivo* community (**Fig. SA2C**). Here, the control diet animals experienced a high within-group variation compared to those given AcGGM supplements, but the phylogenetic diversities32/72 of their gut microbiomes were otherwise similar irrespective of the hosts’ diet.


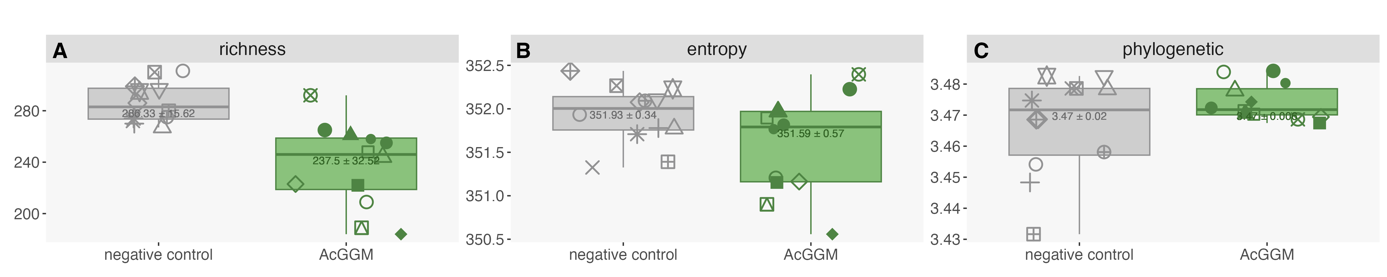


**Figure SA2**. Alpha diversity metrics of the Michalak et al. study ^2^ using samples from pigs fed either negative control or AcGGM-supplemented diets. Numbers overlaying each box denote the mean and standard deviation across the samples of the respective group. The facets display **A)** species richness, measured in counts of unique populations; **B)** Shannon entropy diversity index; and **C)** phylogenetic diversity, based on a pruned version of the phylogenetic tree provided as supplementary data (Suppl. file 4) in ^2^.

We investigated why the InVitSim community appeared narrower with respect to species richness but with an increased phylogenetic diversity in the previous section of this Supplementary file, and found that the taxonomy and functional capacity of taxa with reduced abundance in the *in vitro* gut model were still represented by other community members. This loss of redundancy in the *in vitro* model affecting some of the most prominent gut microbiome members is thus the most likely explanation for the discrepancy between the observed phylogenetic diversity in *in vivo* and InVitSim microbial communities. Still, the concordance of over 70% of taxa retained after fermentation in InVitSim still represents an overwhelming concordance with pre-fermentation samples, and establishes the microbial community model as a good representation of its *in vivo* counterpart.

**Impact of host diet on the in vitro microbial community post-fermentation.**

To investigate the microbial communities inoculated in InVitSim, we conducted differential abundance analyses of all samples – not only from animals fed the control diet during the four-week *in vivo* trail – extracted from the *in vitro* model. Samples from different porcine diet groups revealed prominent changes within the Bacillota phylum, which encompassed 66 of the 78 taxa (>84%) with significantly different abundance across this contrast. Within this phylum, the inclusion of AcGGM fibres in the porcine diet was associated with a shift from Lachnospirales genera like *Bilifractor* (LFC<-3); *Blautia* (LFC<-1); and *Coprococcus* (LFC<-2), to other Clostridia genera like *Butyricicoccus* (LFC>5); *Lachnospira* (LFC>11); *Butyrivibrio* (LFC>9); and *Eubacterium* (LFC>2). Members of the Bacilli genera *Bulleidia* (LFC>1); *Lactobacillus* (LFC>1); and *Limosilactobacillus* (LFC>1) were also found in increased abundance in samples from AcGGM-fed animals (**Fig. SC2**).

Referring to the genome-inferred functional traits for the orders of these taxa (**Fig. 2C**), the observed shift from Lachnospirales to Oscillospirales, Erysipelotrichales, and Lactobacillales following the AcGGM-supplementation of the porcine diet animals is associated with a shift down along the second component axis. The features with the highest weights in this component include “spore”, “SCFA biosynthesis”, and “cellular structure”, suggesting that the InVitSim microbial communities’ theoretical potential for these biological functions was impacted by the presence of AcGGM in the diet of the animal from which they were extracted. Furthermore, 99% of the differentially expressed genes in the taxa with reduced abundance in communities from AcGGM-fed pigs were downregulated. Conversely, almost all genes with differential expression from taxa with increased abundance in AcGGM-fed animals were upregulated. These observations demonstrate that taxa with increased abundance in samples from AcGGM-fed animals also displayed increased transcriptomic levels, indicating that their abundance was due to increased metabolic activity. Few AcGGM-degrading enzymes were among these upregulated genes, but several other CAZymes were found upregulated. These observations suggest that the taxa with increased abundance and transcriptomic activity degrade other types of carbohydrates also present in the diet, like xylan and starch, or act as secondary degraders of the AcGGM fibre.

**Optical density measurements**


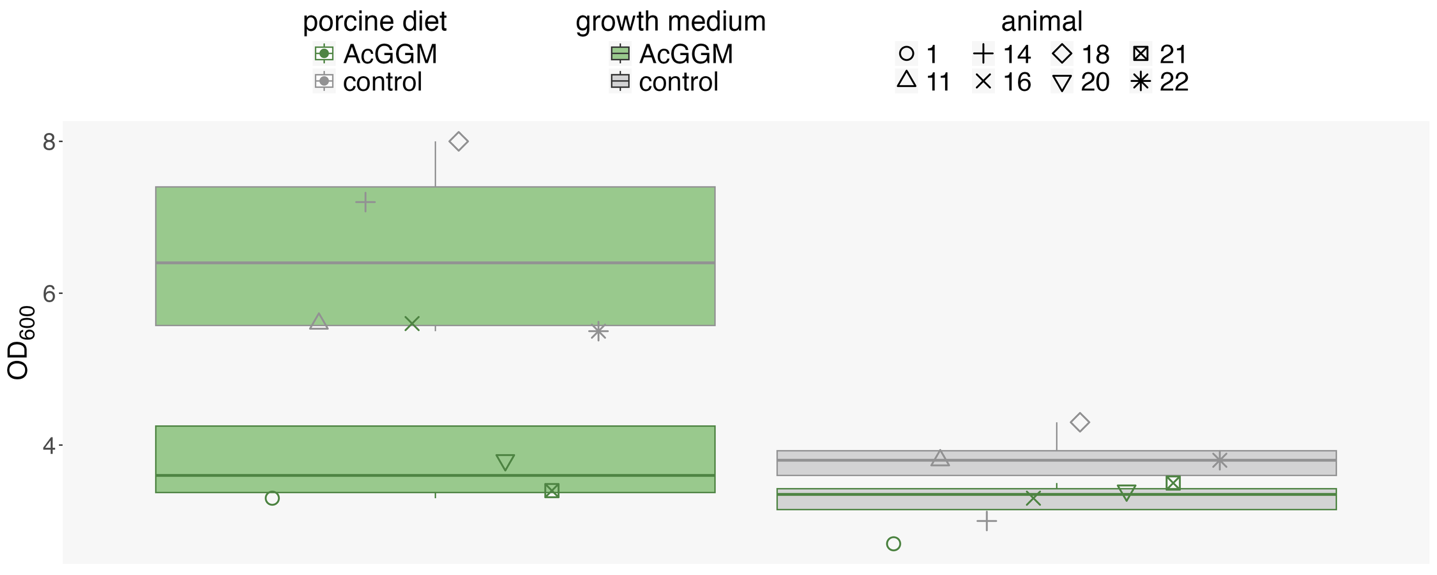


**Figure SA3**. Optical density measurements of samples of different porcine diet and growth medium groups. Boxplot fill reflects whether samples were inoculated in fermentors with (green) or without (grey) AcGGM added as a carbon source. Point colour indicate the diet group of the donor animal.

1. Sebastià C, Folch JM, Ballester M, Estellé J, Passols M, Muñoz M, García-Casco JM, Fernández AI, Castelló A, Sánchez A, Crespo-Piazuelo D. 2024. Interrelation between gut microbiota, SCFA, and fatty acid composition in pigs. mSystems 9:e01049-23. [↑](#footnote-ref-2)
2. Michalak L, Gaby JC, Lagos L, La Rosa SL, Hvidsten TR, Tétard-Jones C, Willats WGT, Terrapon N, Lombard V, Henrissat B, Dröge J, Arntzen MØ, Hagen LH, Øverland M, Pope PB, Westereng B. 2020. Microbiota-directed fibre activates both targeted and secondary metabolic shifts in the distal gut. Nat Commun 11:5773. [↑](#footnote-ref-3)
