# Supplementary material for: In vitro model reveals structural and metabolic insights into the porcine cecal microbiota in response to β-mannan exposure
Source: Appl Environ Microbiol. 2026 Jun 18;92(7):e00140-26. doi: 10.1128/aem.00140-26 (PMC13390407; doi:10.1128/aem.00140-26)
Supplement: Supplemental File B — Visualizations of the abundances of populations summed at the genus level and shown as individual populations on the species level. [file aem.00140-26-s0002.pdf]

Supplementary file B

In vitro model reveals structural and metabolic insights to the porcine caecal microbiota in response to β-mannan exposure

This supplementary contains additional visualisations of the abundances of populations found in the metagenome-assembled genome catalogues of both the present *in vitro* and the 2020 *in vivo* study by Michalak *et al*. [1]. The abundances are summed at the genus-level (**Fig. SB1**) and shown as individual populations on the species level (**Fig. SB2**).

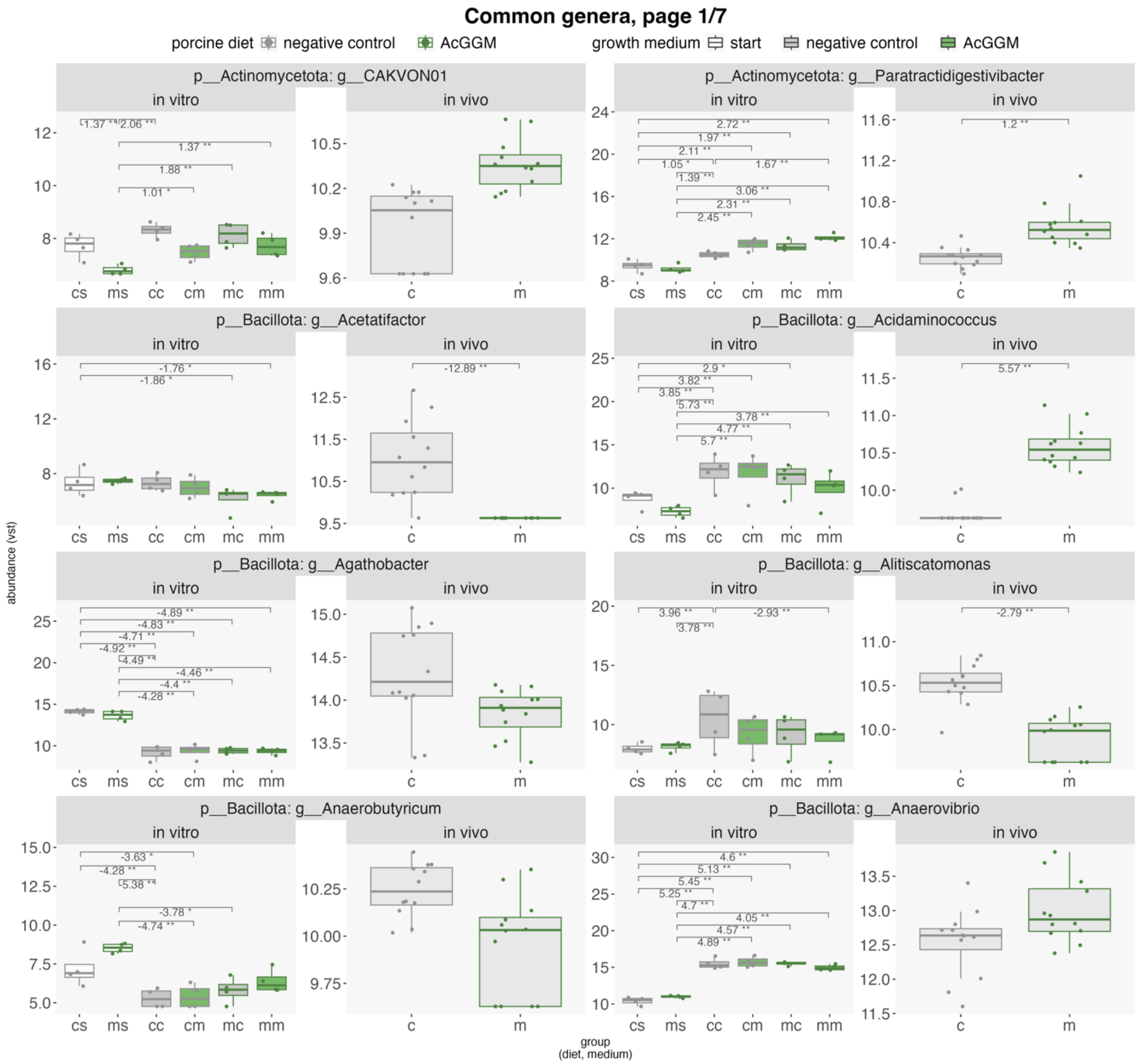

***Figure SB1**. Variance-stabilised abundances of microbial populations at the genus-level of common taxa between the present in vitro study and the 2020 in vivo study by Michalak et al.* [1]*, set 1 of 7. Significant differential abundances between pairs of boxplots are indicated by horizontal bars with accompanying log2 fold change (thresholds ||LFC|| > 1 and base mean > 50) and FDR-adjusted p-values indicated by asterisks (* < 0.05, ** < 0.01, *** 0.001).*

---

[1] Michalak L, Gaby JC, Lagos L, La Rosa SL, Hvidsten TR, Tétard-Jones C, Willats WGT, Terrapon N, Lombard V, Henrissat B, Dröge J, Arntzen MØ, Hagen LH, Øverland M, Pope PB, Westereng B. 2020. Microbiota-directed fibre activates both targeted and secondary metabolic shifts in the distal gut. Nat Commun 11:5773.

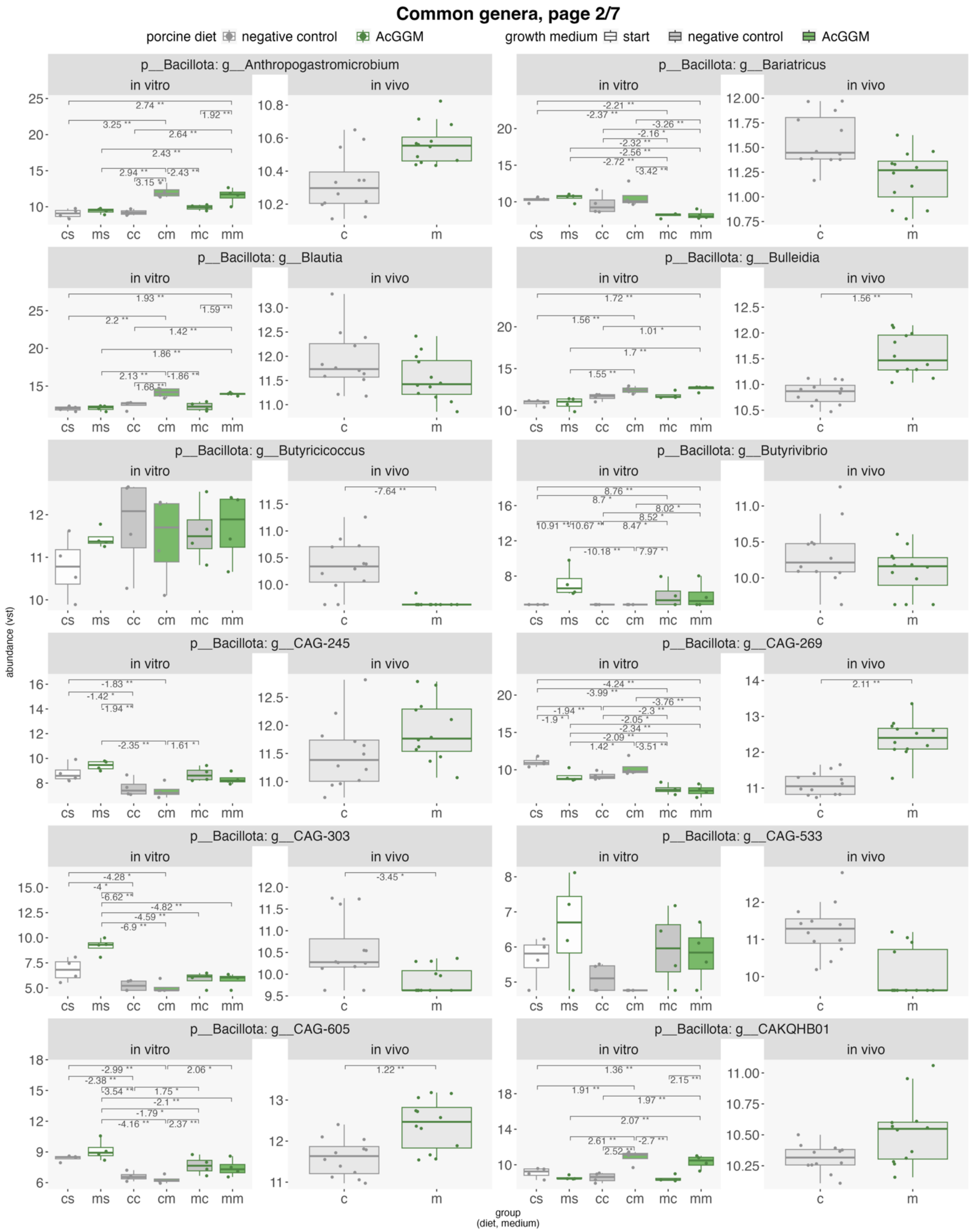

Figure SB1 continued. *Variance-stabilised abundances of microbial populations at the genus-level of common taxa between the present in vitro study and the 2020 in vivo study by Michalak et al.* [1]*, set 2 of 7. Significant differential abundances between pairs of boxplots are indicated by horizontal bars with accompanying log2 fold change (thresholds ||LFC|| > 1 and base mean > 50) and FDR-adjusted p-values indicated by asterisks (* < 0.05, ** < 0.01, *** 0.001).*

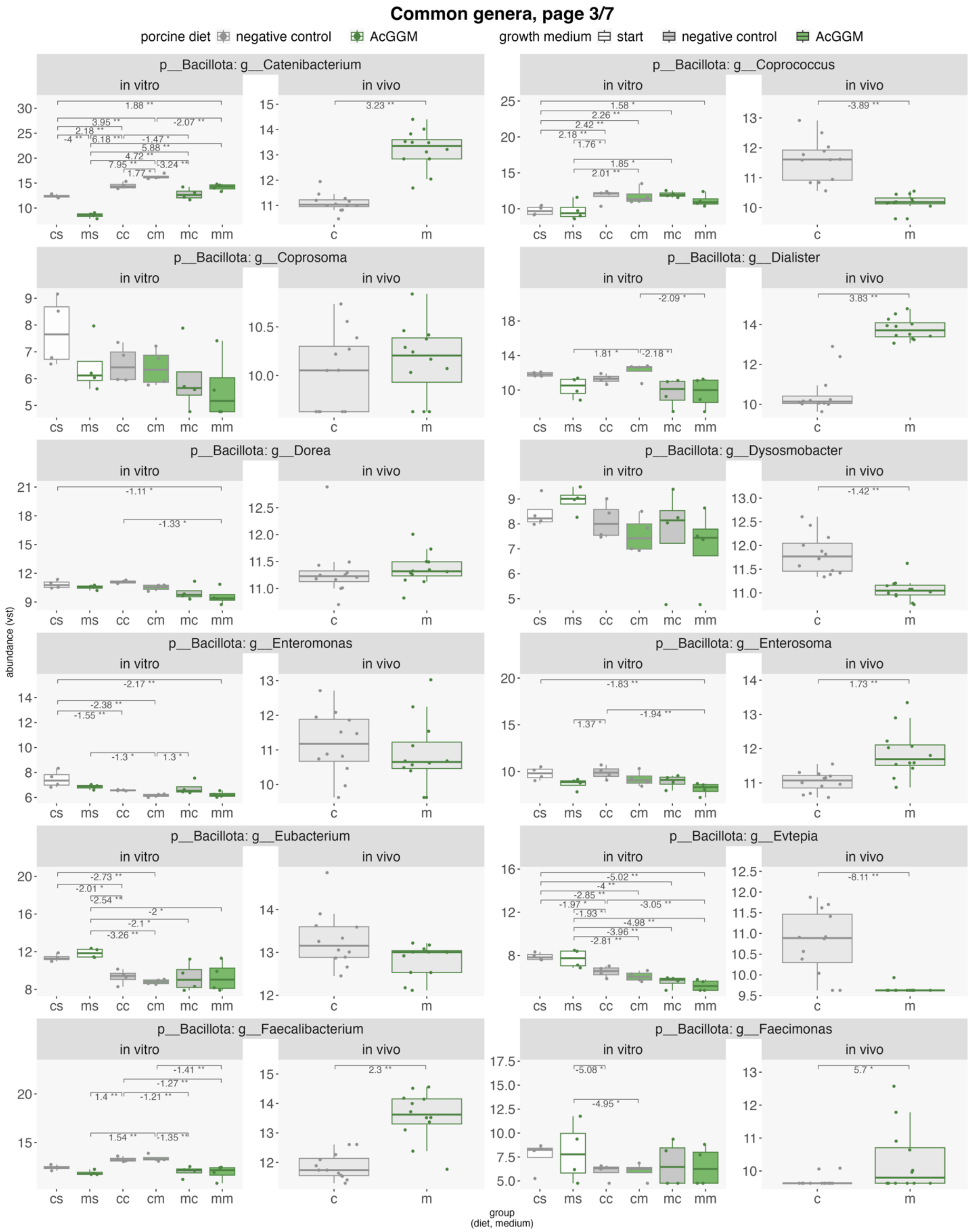

***Figure SB1 continued**. Variance-stabilised abundances of microbial populations at the genus-level of common taxa between the present in vitro study and the 2020 in vivo study by Michalak et al. [1], set 3 of 7. Significant differential abundances between pairs of boxplots are indicated by horizontal bars with accompanying log2 fold change (thresholds ||LFC|| > 1 and base mean > 50) and FDR-adjusted p-values indicated by asterisks (* < 0.05, ** < 0.01, *** 0.001).*

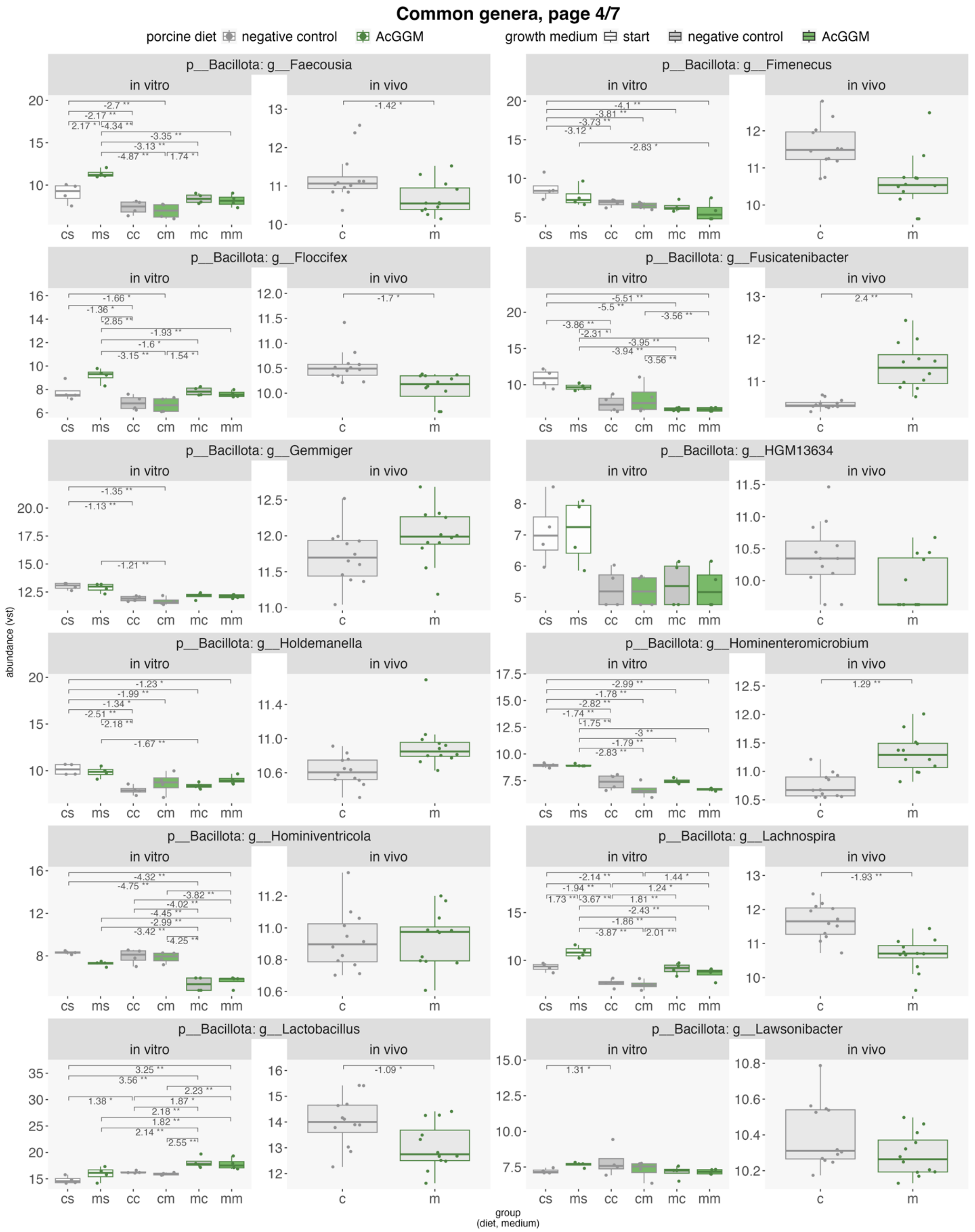

Figure SB1 continued. *Variance-stabilised abundances of microbial populations at the genus-level of common taxa between the present in vitro study and the 2020 in vivo study by Michalak et al. [1], set 4 of 7. Significant differential abundances between pairs of boxplots are indicated by horizontal bars with accompanying log2 fold change (thresholds ||LFC|| > 1 and base mean > 50) and FDR-adjusted p-values indicated by asterisks (* < 0.05, ** < 0.01, *** 0.001).*

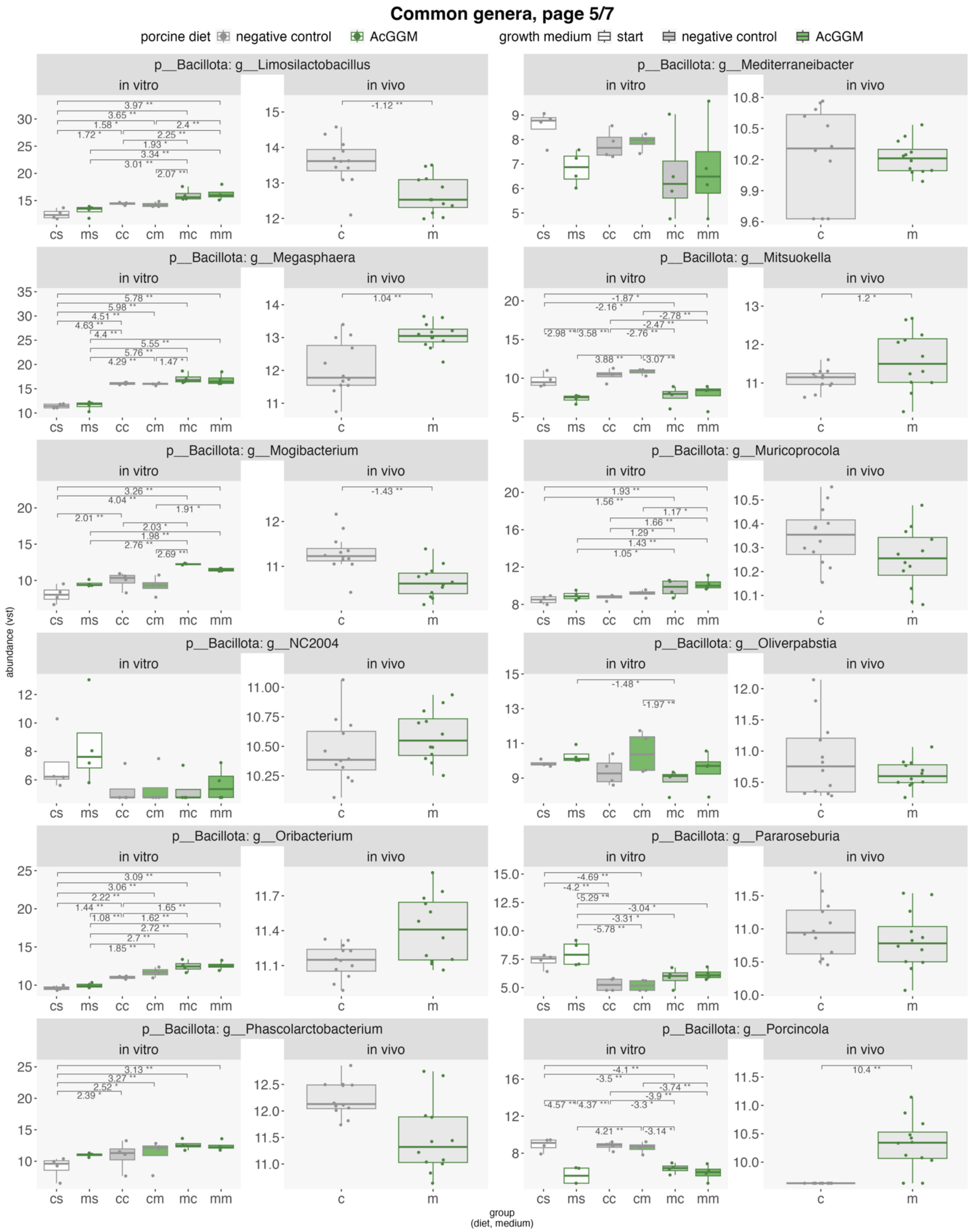

Figure SB1 continued. *Variance-stabilised abundances of microbial populations at the genus-level of common taxa between the present in vitro study and the 2020 in vivo study by Michalak et al. [1], set 5 of 7. Significant differential abundances between pairs of boxplots are indicated by horizontal bars with accompanying log2 fold change (thresholds ||LFC|| > 1 and base mean > 50) and FDR-adjusted p-values indicated by asterisks (* < 0.05, ** < 0.01, *** 0.001).*

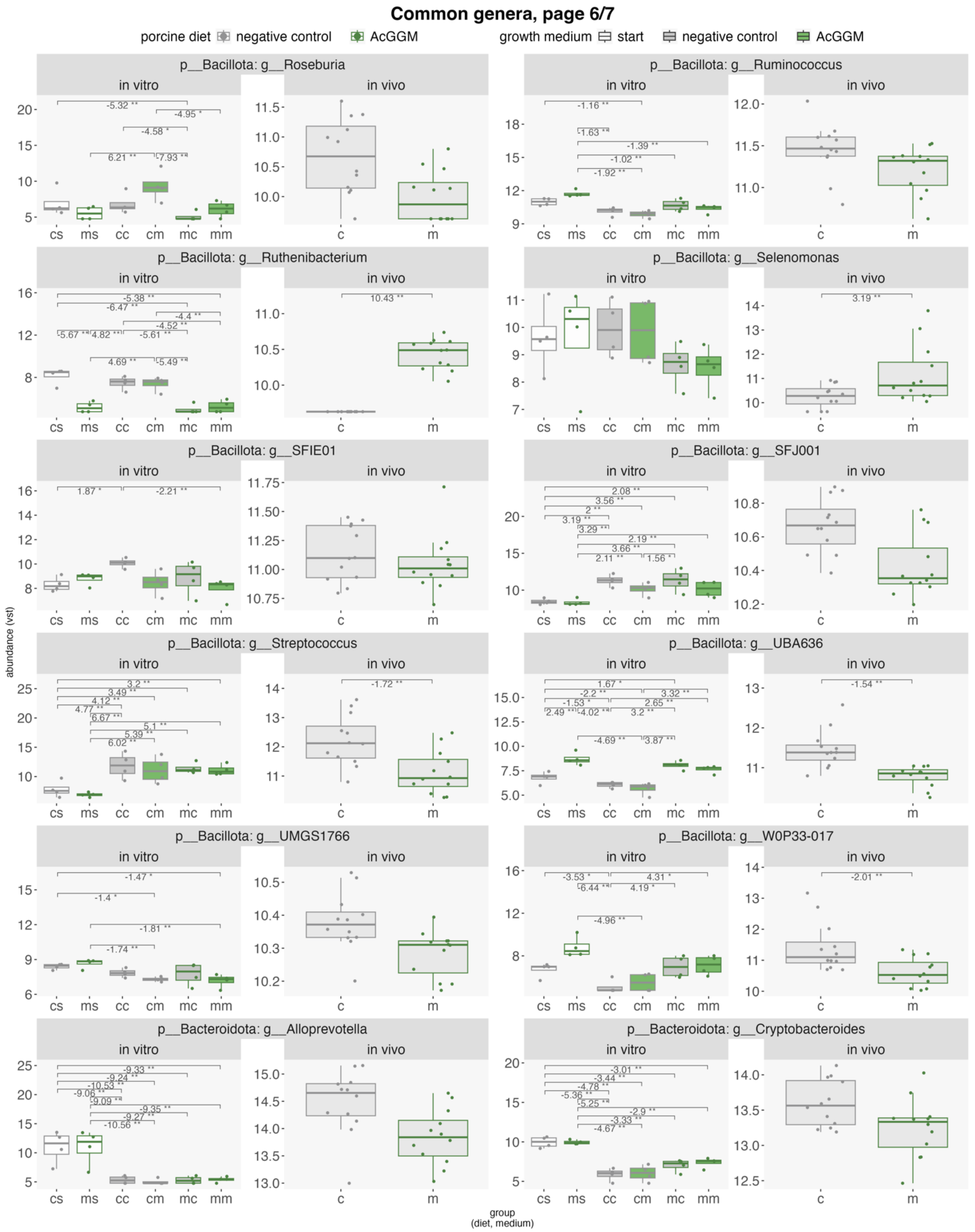

***Figure SB1 continued**. Variance-stabilised abundances of microbial populations at the genus-level of common taxa between the present in vitro study and the 2020 in vivo study by Michalak et al. [1], set 6 of 7. Significant differential abundances between pairs of boxplots are indicated by horizontal bars with accompanying log2 fold change (thresholds ||LFC|| > 1 and base mean > 50) and FDR-adjusted p-values indicated by asterisks (* < 0.05, ** < 0.01, *** 0.001).*

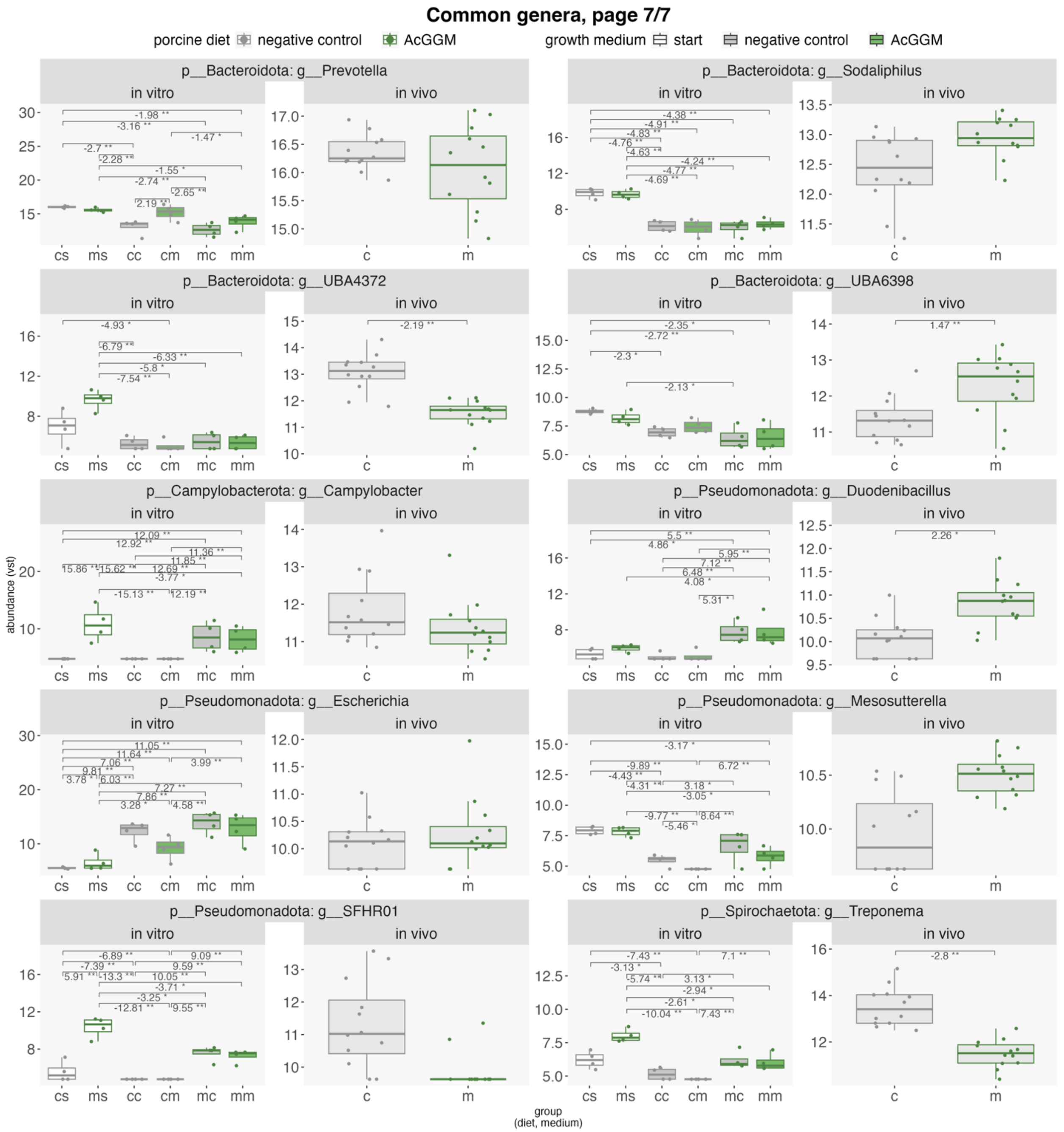

***Figure SB1 continued**. Variance-stabilised abundances of microbial populations at the genus-level of common taxa between the present in vitro study and the 2020 in vivo study by Michalak et al. [1], set 7 of 7. Significant differential abundances between pairs of boxplots are indicated by horizontal bars with accompanying log2 fold change (thresholds ||LFC|| > 1 and base mean > 50) and FDR-adjusted p-values indicated by asterisks (* < 0.05, ** < 0.01, *** 0.001).*

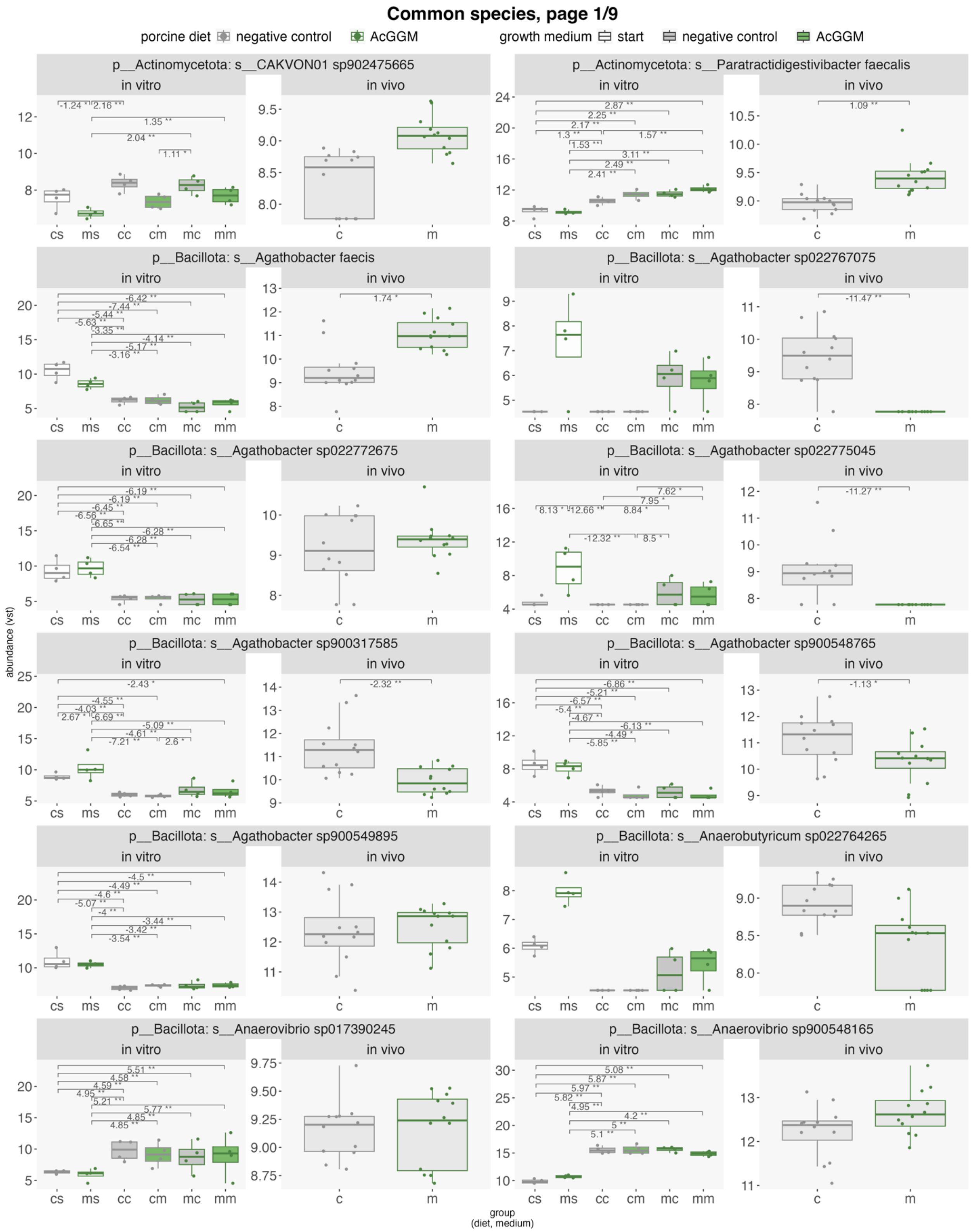

Figure SB2. *Variance-stabilised abundances of microbial populations at the species-level of common taxa between the present in vitro study and the 2020 in vivo study by Michalak et al.* [1]*, set 1 of 9. Significant differential abundances between pairs of boxplots are indicated by horizontal bars with accompanying log2 fold change (thresholds ||LFC|| > 1 and base mean > 50) and FDR-adjusted p-values indicated by asterisks (* < 0.05, ** < 0.01, *** 0.001).*

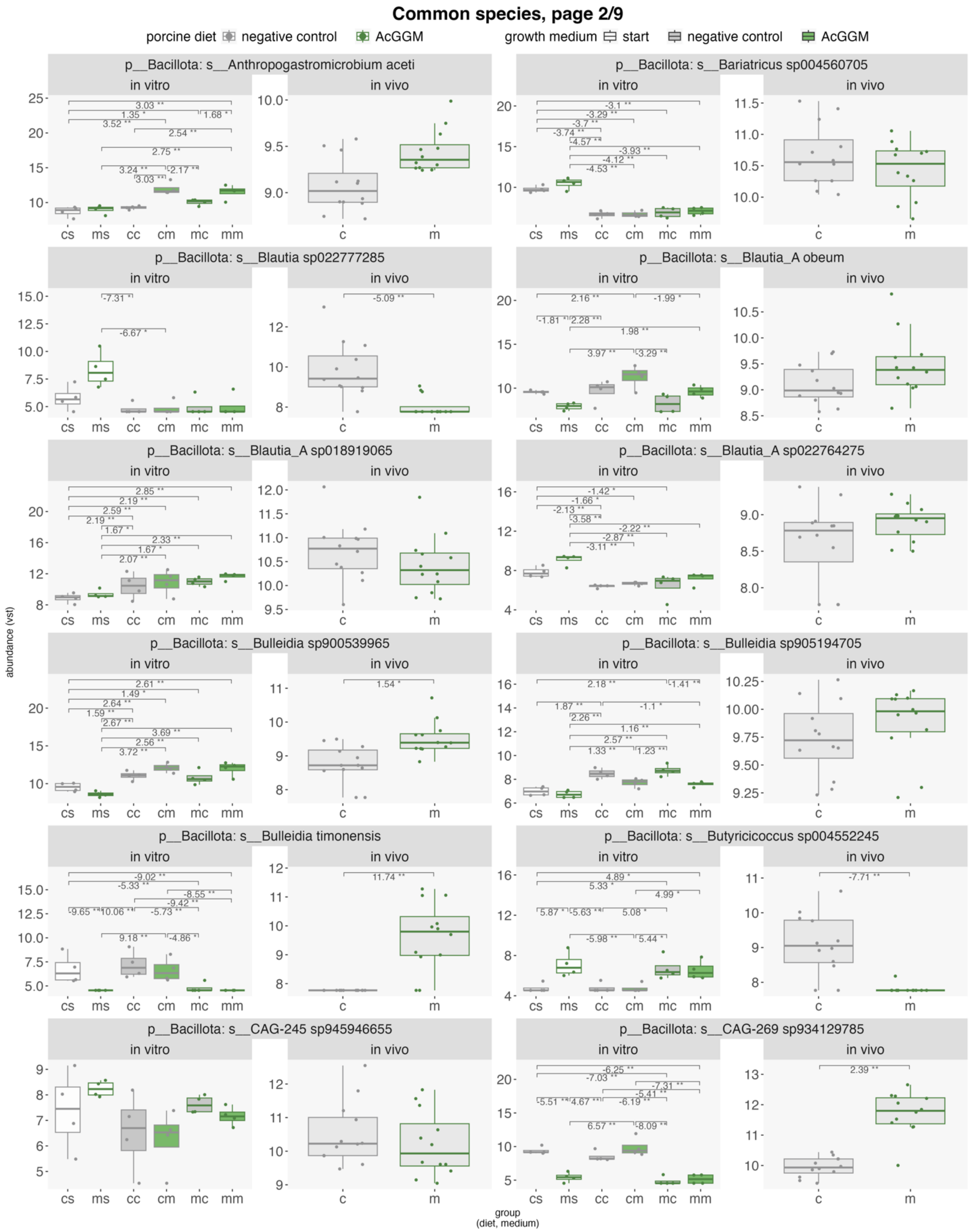

Figure SB2 continued. *Variance-stabilised abundances of microbial populations at the species-level of common taxa between the present in vitro study and the 2020 in vivo study by Michalak et al. [1], set 2 of 9. Significant differential abundances between pairs of boxplots are indicated by horizontal bars with accompanying log2 fold change (thresholds ||LFC|| > 1 and base mean > 50) and FDR-adjusted p-values indicated by asterisks (* < 0.05, ** < 0.01, *** 0.001).*

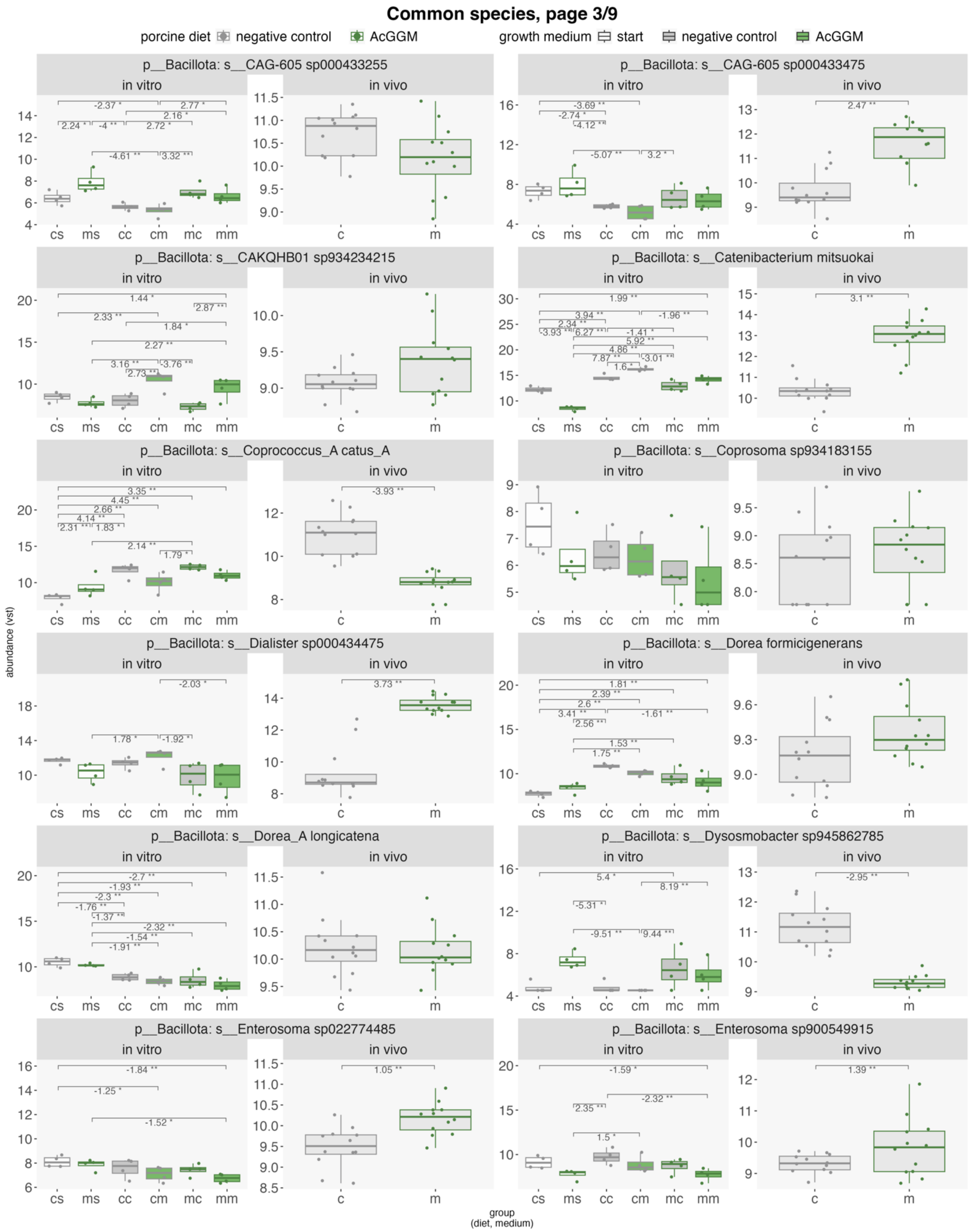

Figure SB2 continued. *Variance-stabilised abundances of microbial populations at the species-level of common taxa between the present in vitro study and the 2020 in vivo study by Michalak et al. [1], set 3 of 9. Significant differential abundances between pairs of boxplots are indicated by horizontal bars with accompanying log2 fold change (thresholds ||LFC|| > 1 and base mean > 50) and FDR-adjusted p-values indicated by asterisks (* < 0.05, ** < 0.01, *** 0.001).*

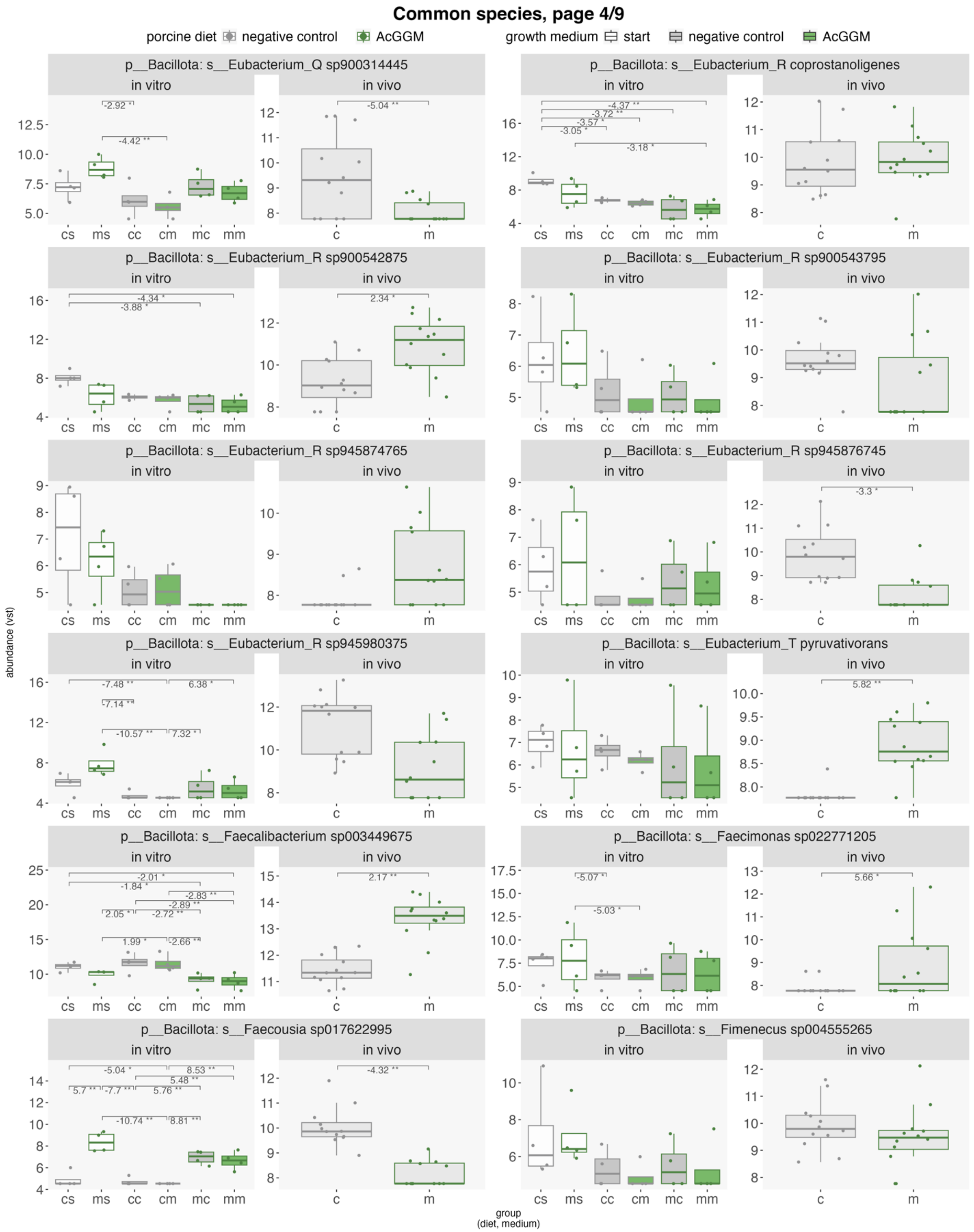

Figure SB2 continued. *Variance-stabilised abundances of microbial populations at the species-level of common taxa between the present in vitro study and the 2020 in vivo study by Michalak et al.* [1]*, set 4 of 9. Significant differential abundances between pairs of boxplots are indicated by horizontal bars with accompanying log2 fold change (thresholds ||LFC|| > 1 and base mean > 50) and FDR-adjusted p-values indicated by asterisks (* < 0.05, ** < 0.01, *** 0.001).*

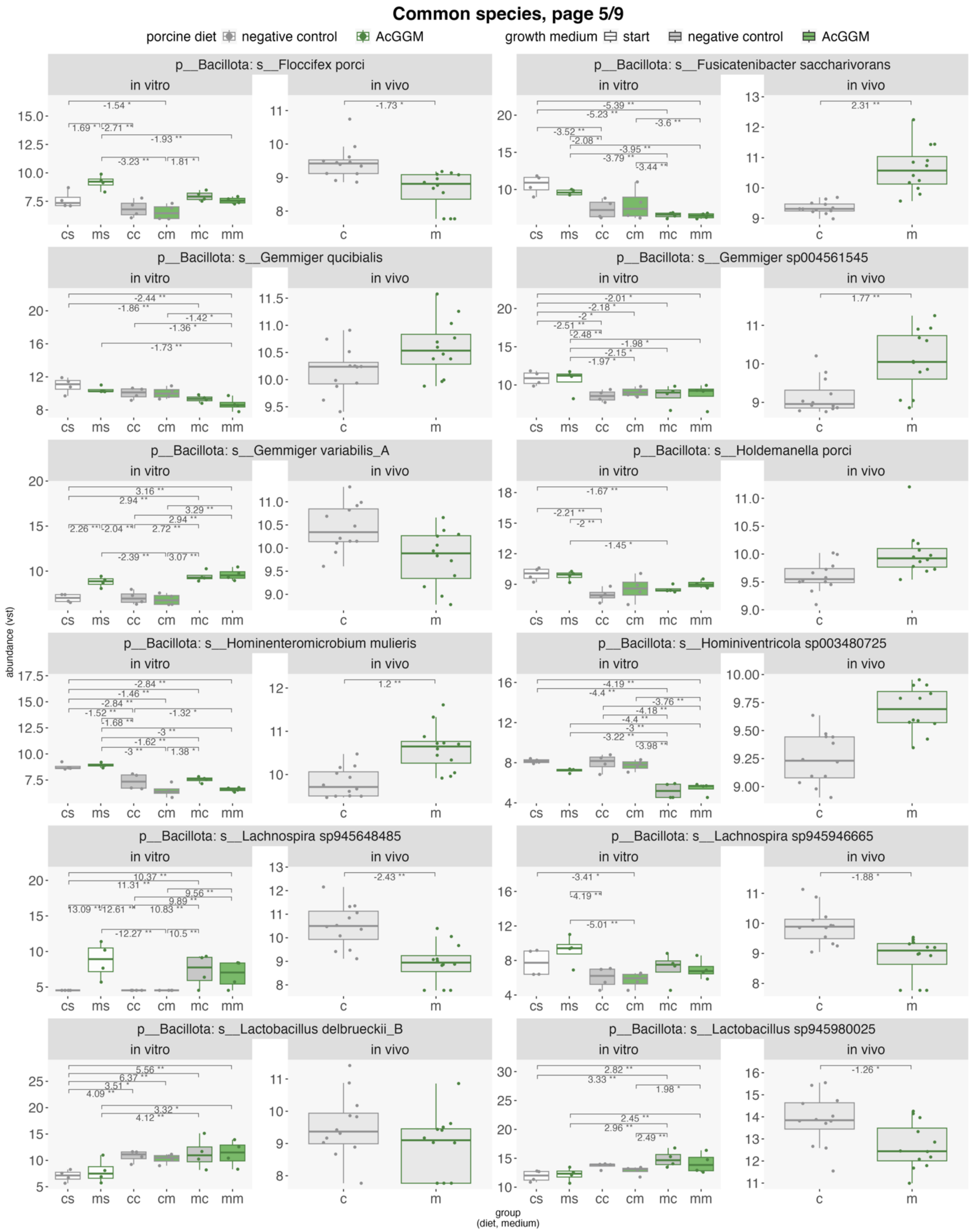

Figure SB2 continued*. Variance-stabilised abundances of microbial populations at the species-level of common taxa between the present in vitro study and the 2020 in vivo study by Michalak et al. [1], set 5 of 9. Significant differential abundances between pairs of boxplots are indicated by horizontal bars with accompanying log2 fold change (thresholds ||LFC|| > 1 and base mean > 50) and FDR-adjusted p-values indicated by asterisks (* < 0.05, ** < 0.01, *** 0.001).*

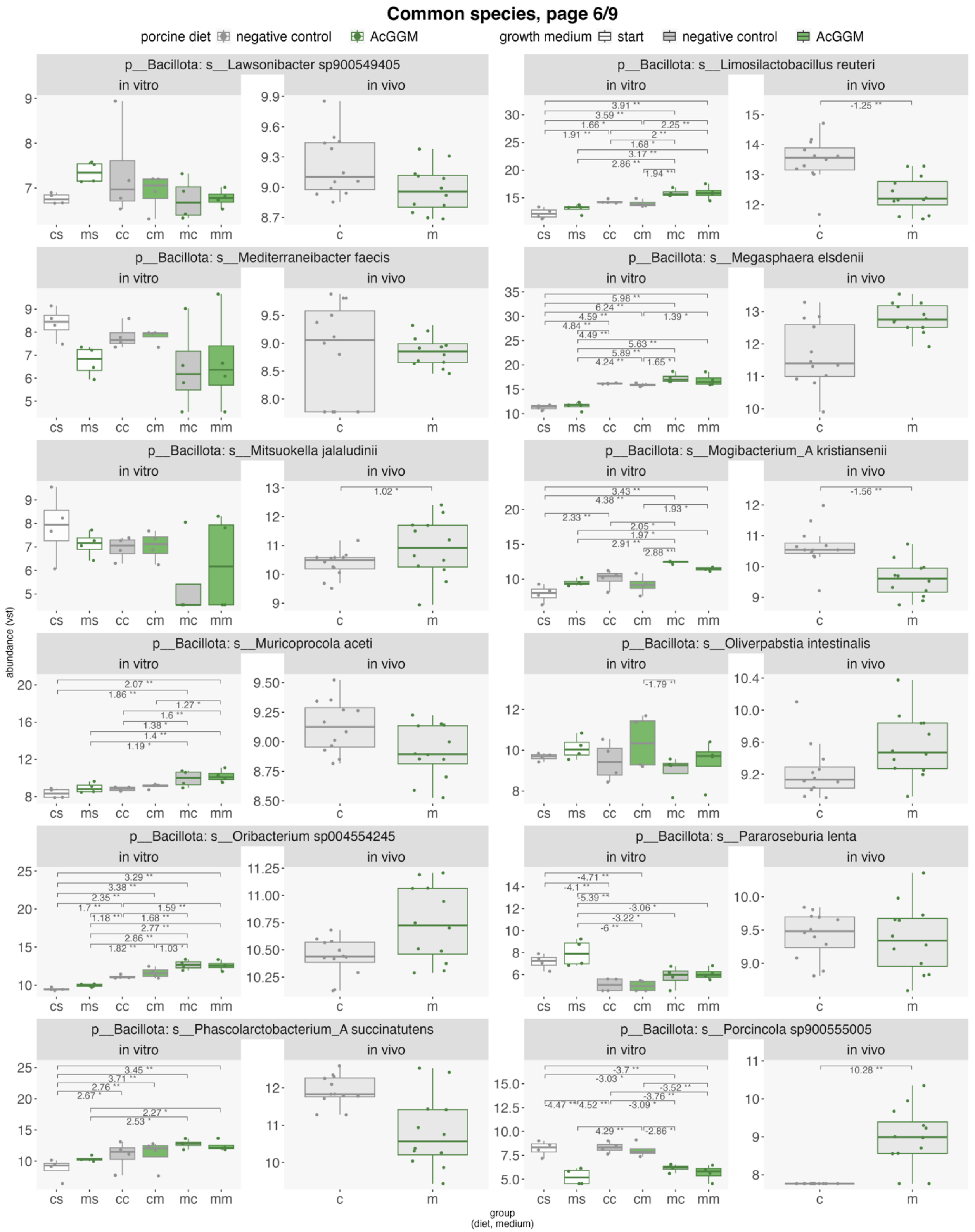

Figure SB2 continued. *Variance-stabilised abundances of microbial populations at the species-level of common taxa between the present in vitro study and the 2020 in vivo study by Michalak et al. [1], set 6 of 9. Significant differential abundances between pairs of boxplots are indicated by horizontal bars with accompanying log2 fold change (thresholds ||LFC|| > 1 and base mean > 50) and FDR-adjusted p-values indicated by asterisks (* < 0.05, ** < 0.01, *** 0.001).*

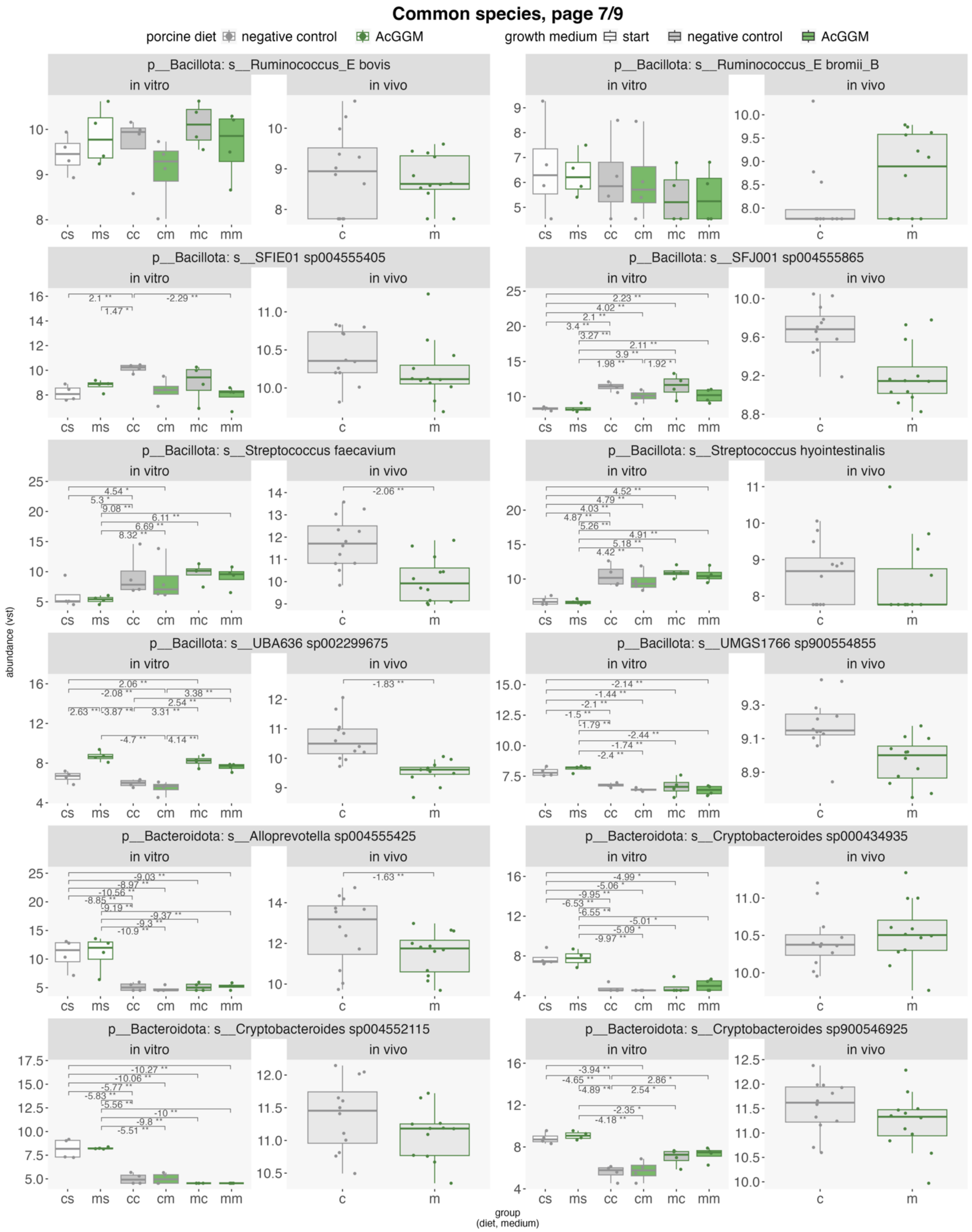

Figure SB2 continued. *Variance-stabilised abundances of microbial populations at the species-level of common taxa between the present in vitro study and the 2020 in vivo study by Michalak et al. [1], set 7 of 9. Significant differential abundances between pairs of boxplots are indicated by horizontal bars with accompanying log2 fold change (thresholds ||LFC|| > 1 and base mean > 50) and FDR-adjusted p-values indicated by asterisks (* < 0.05, ** < 0.01, *** 0.001).*

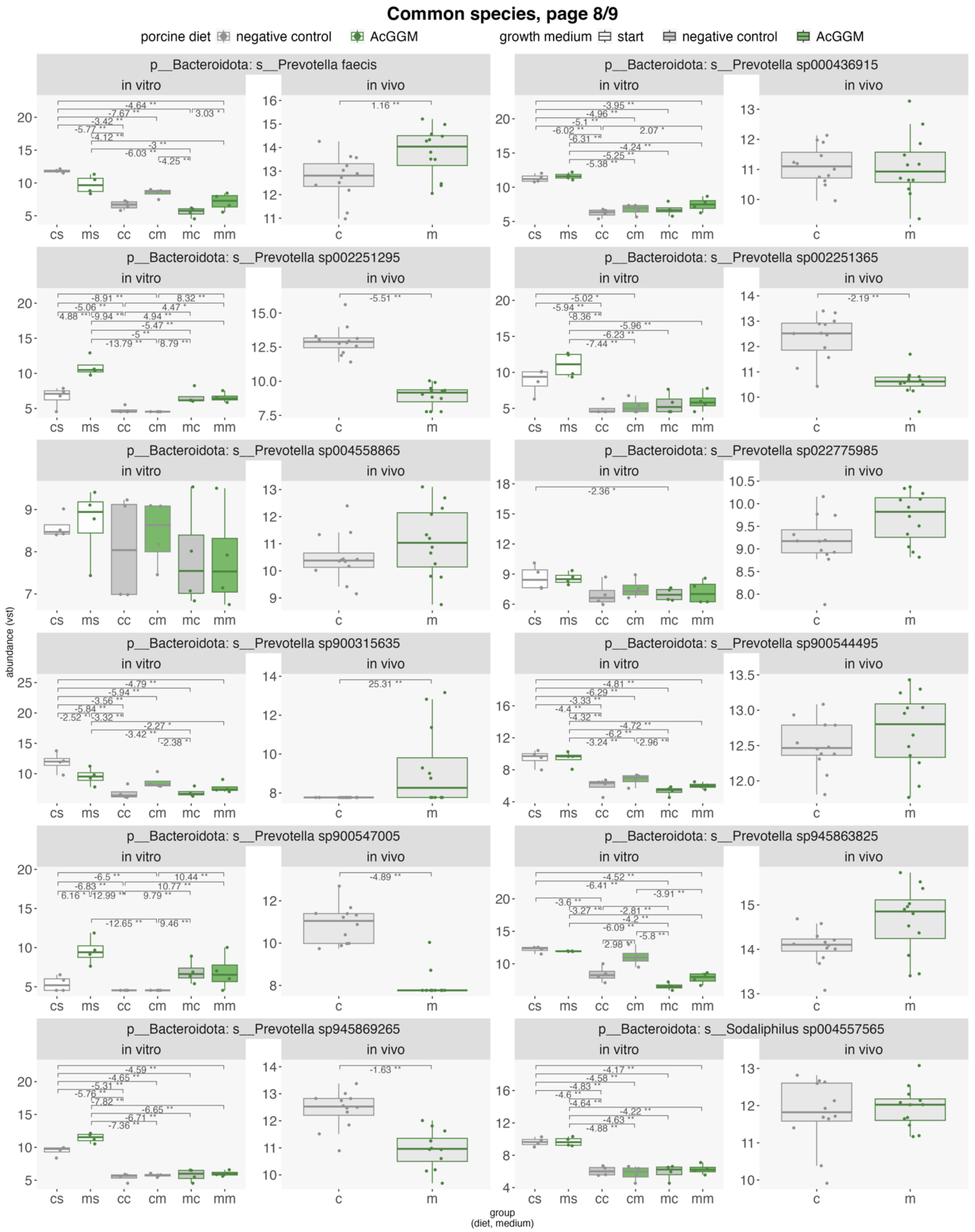

Figure SB2 continued. *Variance-stabilised abundances of microbial populations at the species-level of common taxa between the present in vitro study and the 2020 in vivo study by Michalak et al.* [1]*, set 8 of 9. Significant differential abundances between pairs of boxplots are indicated by horizontal bars with accompanying log2 fold change (thresholds ||LFC|| > 1 and base mean > 50) and FDR-adjusted p-values indicated by asterisks (* < 0.05, ** < 0.01, *** 0.001).*

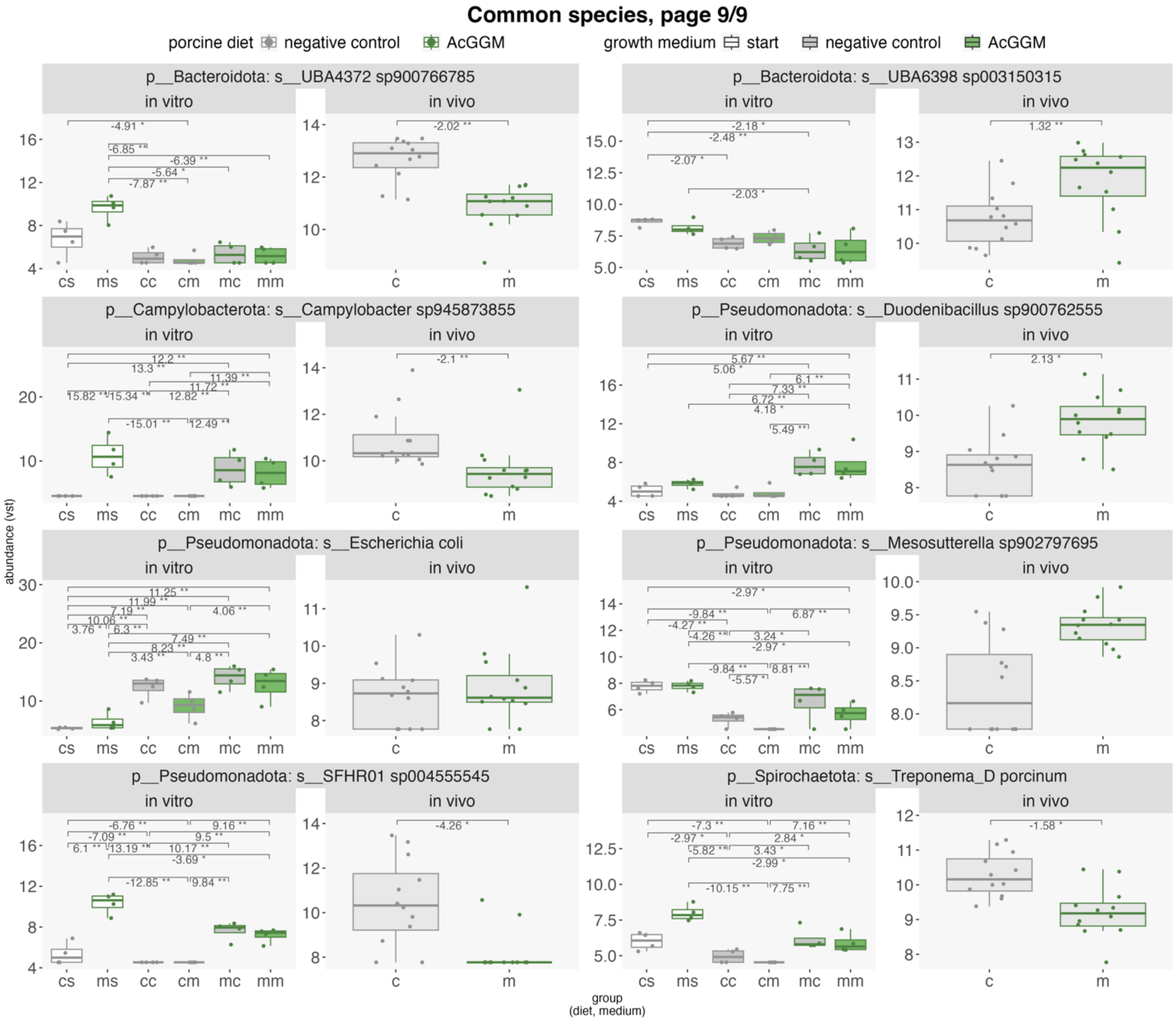

Figure SB2 continued. *Variance-stabilised abundances of microbial populations at the species-level of common taxa between the present in vitro study and the 2020 in vivo study by Michalak et al. [1], set 9 of 9. Significant differential abundances between pairs of boxplots are indicated by horizontal bars with accompanying log2 fold change (thresholds ||LFC|| > 1 and base mean > 50) and FDR-adjusted p-values indicated by asterisks (* < 0.05, ** < 0.01, *** 0.001).*
