# Supplementary material for: In vitro model reveals structural and metabolic insights into the porcine cecal microbiota in response to β-mannan exposure
Source: Appl Environ Microbiol. 2026 Jun 18;92(7):e00140-26. doi: 10.1128/aem.00140-26 (PMC13390407; doi:10.1128/aem.00140-26)
Supplement: Supplemental File C — Differential abundance analysis result visualizations. [file aem.00140-26-s0003.pdf]

Supplementary file C

In vitro model reveals structural and metabolic insights to the porcine caecal microbiota in response to β-mannan exposure

All results of the differential abundance and expression analyses are reported in separate files uploaded to the GitHub repository of the manuscript (jennymerkesvik/3domics_wp7_in-vitro-fermentation).

This supplement contains visualisations of the metagenome-assembled genomes with significant abundance difference (||LFC|| > 1, FDR p-value < 0.05, base mean > 50) across one of the study's contrasts. The populations were compared across sampling time (**Fig. SC1**), porcine diet (**Fig. SC2**), growth medium (**Fig. SC3**), and a combined diet and medium contrast group (**Fig. SC4**).

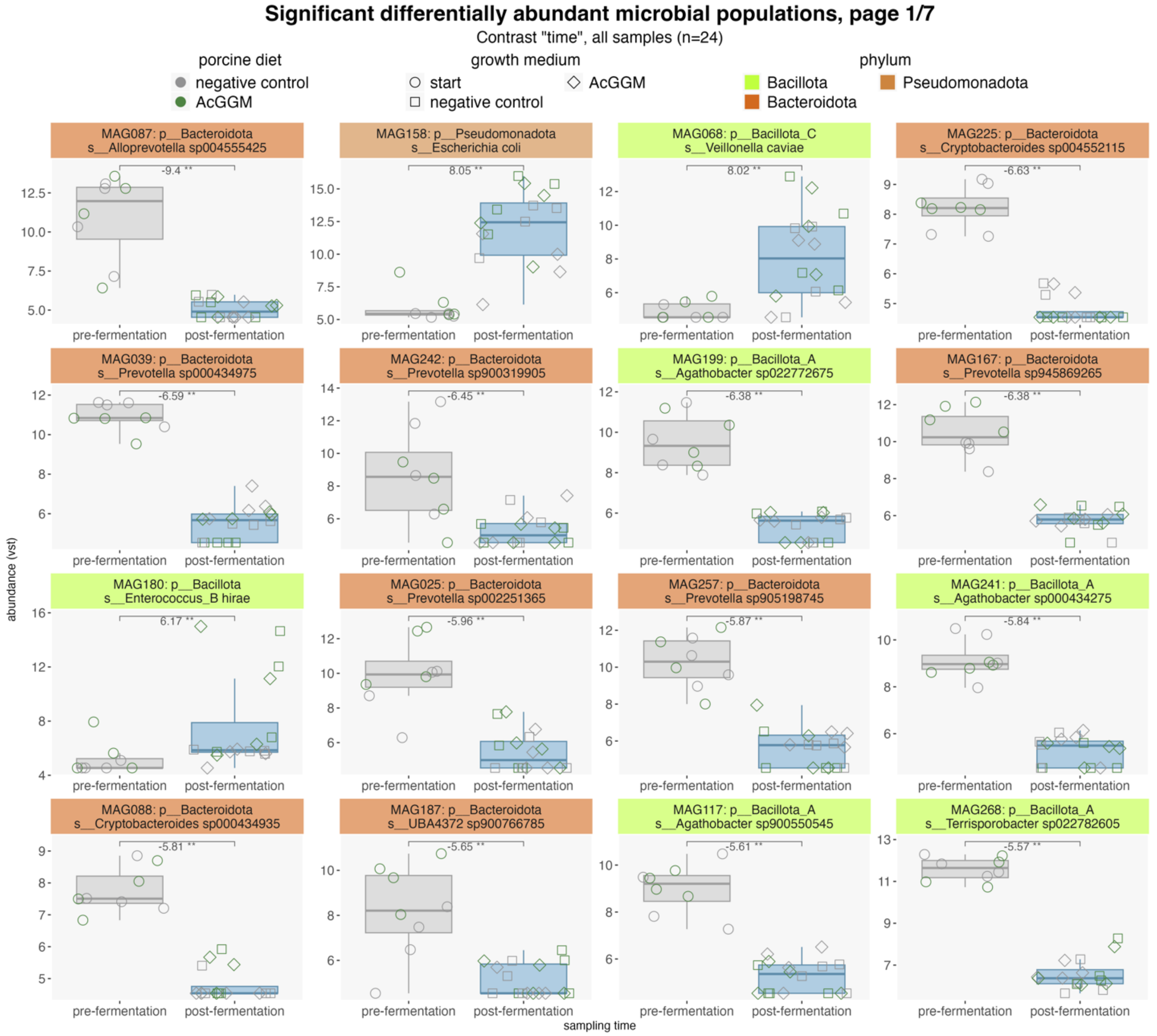

***Figure SC1**. Variance-stabilised abundances of microbial populations with significant abundance changes over time, sorted by largest absolute log2 fold change, set 1 of 7. Significance is indicated with horizontal bars with accompanying log2 fold change (thresholds ||LFC|| > 1 and base mean > 50) and FDR-adjusted p-values indicated by asterisks (* < 0.05, ** < 0.01, *** 0.001).*

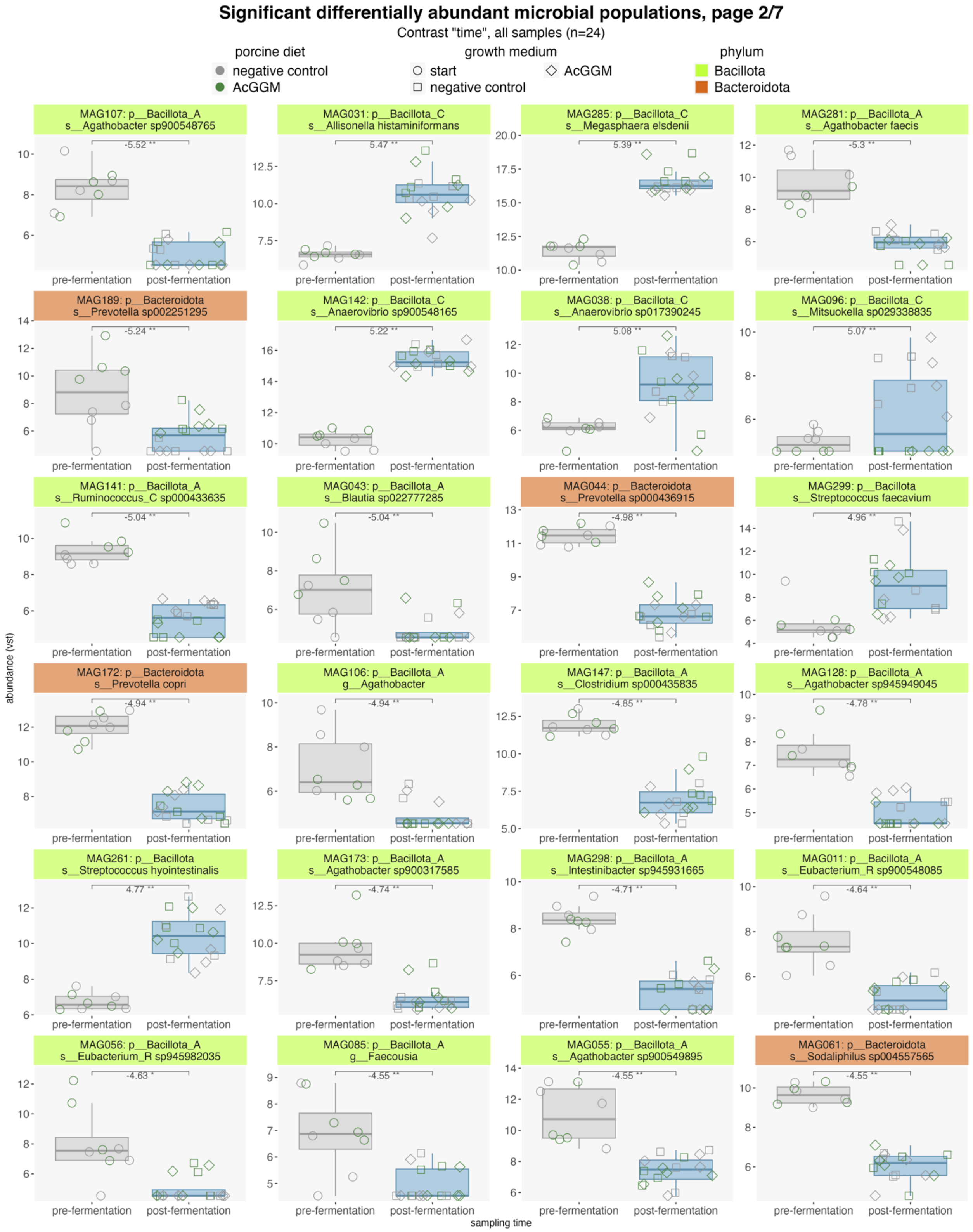

***Figure SC1 continued**. Variance-stabilised abundances of microbial populations with significant abundance changes over time, sorted by largest absolute log2 fold change, set 2 of 7. Significance is indicated with horizontal bars with accompanying log2 fold change (thresholds ||LFC|| > 1 and base mean > 50) and FDR-adjusted p-values indicated by asterisks (* < 0.05, ** < 0.01, *** 0.001).*

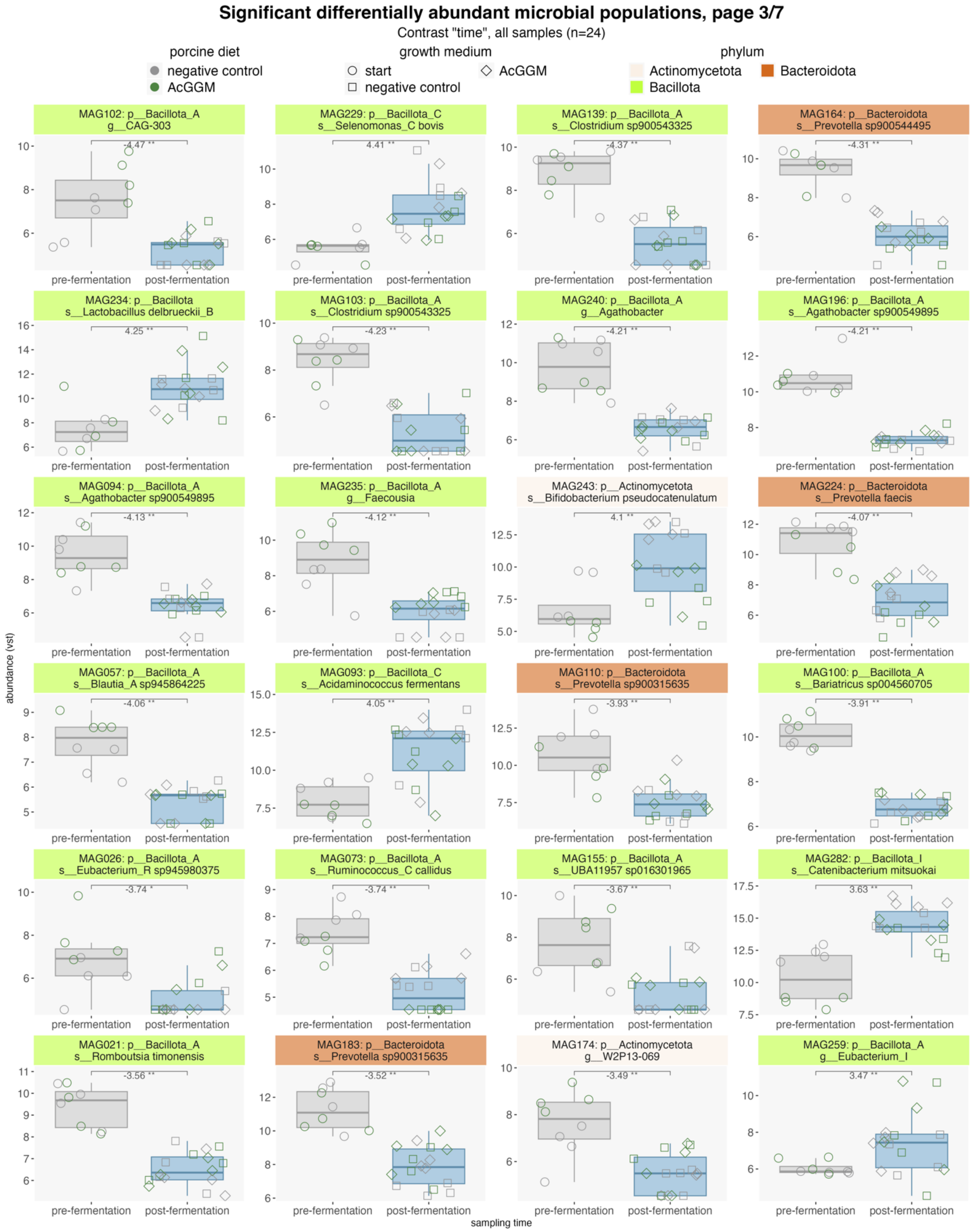

Figure SC1 continued*. Variance-stabilised abundances of microbial populations with significant abundance changes over time, sorted by largest absolute log2 fold change, set 3 of 7. Significance is indicated with horizontal bars with accompanying log2 fold change (thresholds ||LFC|| > 1 and base mean > 50) and FDR-adjusted p-values indicated by asterisks (* < 0.05, ** < 0.01, *** 0.001).*

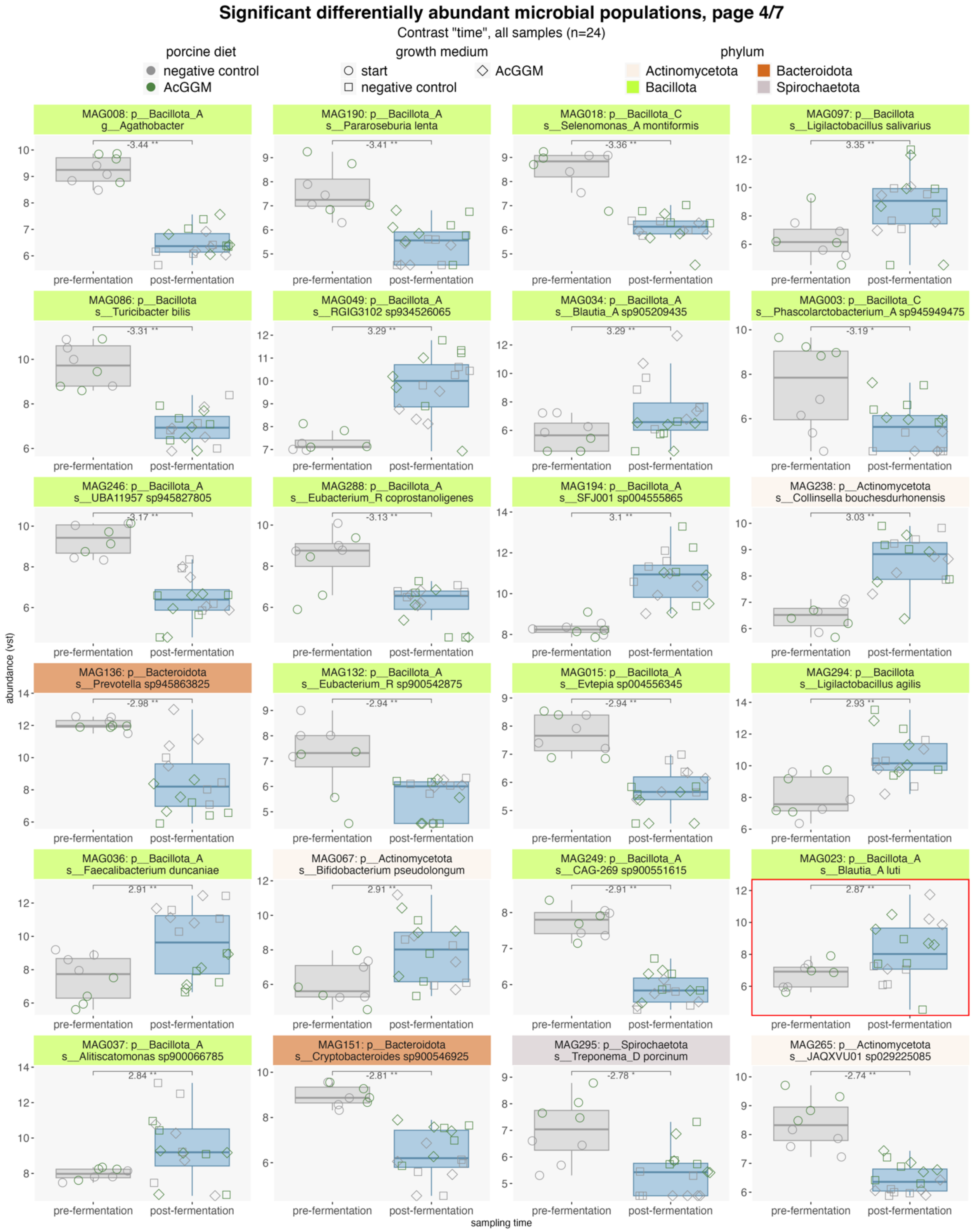

Figure SC1 continued. *Variance-stabilised abundances of microbial populations with significant abundance changes over time, sorted by largest absolute log2 fold change, set 4 of 7. Significance is indicated with horizontal bars with accompanying log2 fold change (thresholds ||LFC|| > 1 and base mean > 50) and FDR-adjusted p-values indicated by asterisks (* < 0.05, ** < 0.01, *** 0.001). Populations suggested to be new AcGGM degraders have been highlighted with a red frame.*

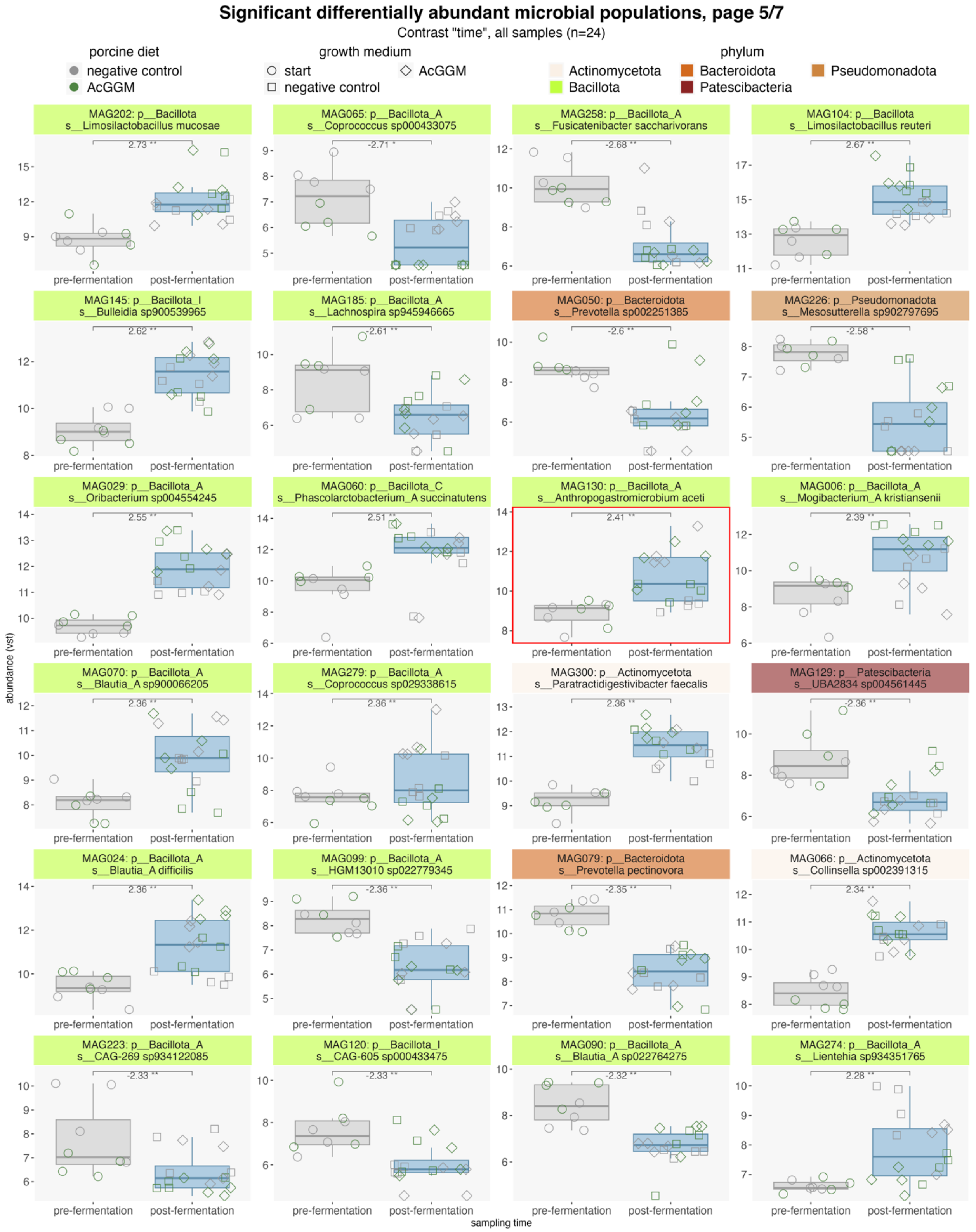

***Figure SC1 continued**. Variance-stabilised abundances of microbial populations with significant abundance changes over time, sorted by largest absolute log2 fold change, set 5 of 7. Significance is indicated with horizontal bars with accompanying log2 fold change (thresholds ||LFC|| > 1 and base mean > 50) and FDR-adjusted p-values indicated by asterisks (* < 0.05, ** < 0.01, *** 0.001). Populations suggested to be new AcGGM degraders have been highlighted with a red frame.*

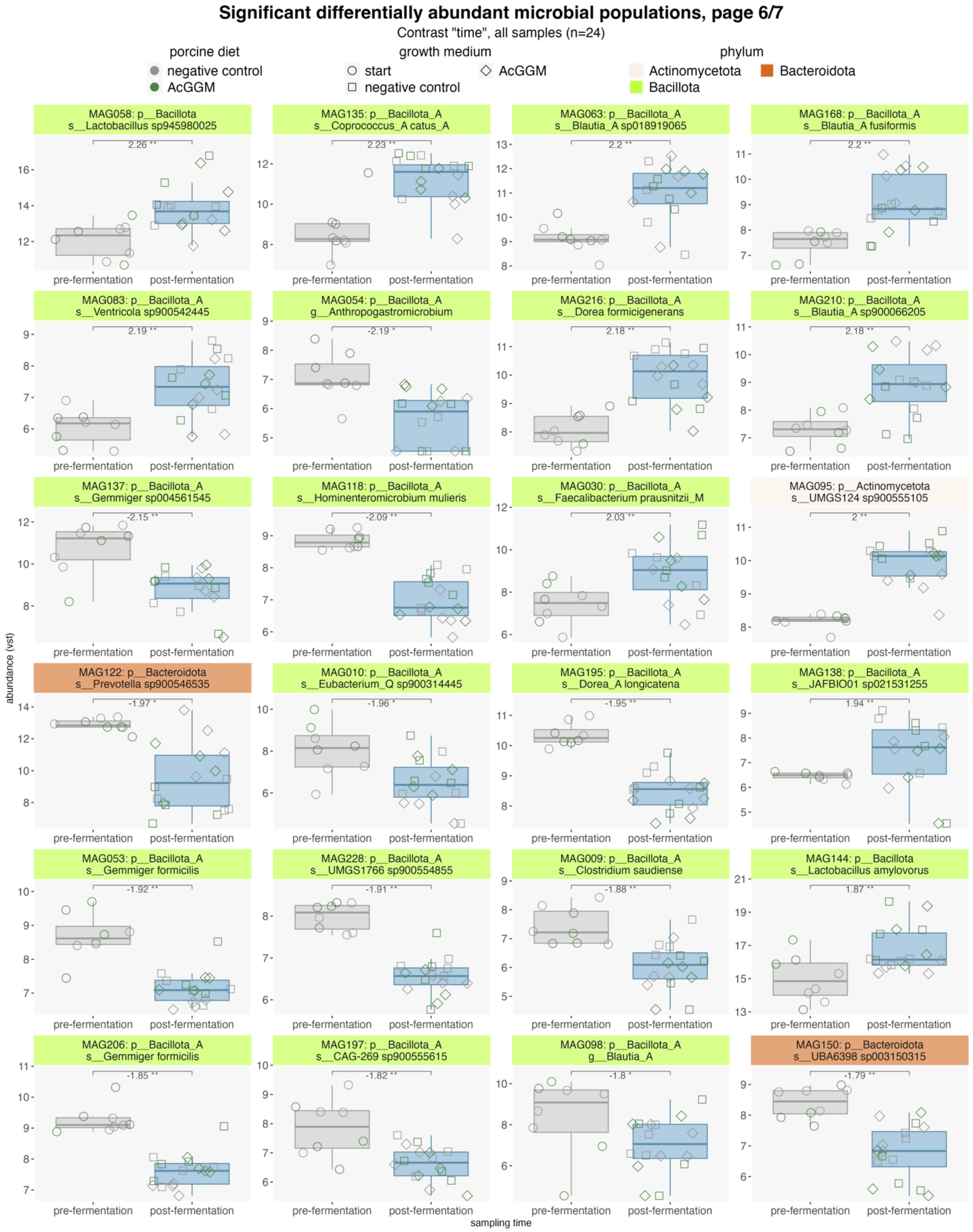

Figure SC1 continued. *Variance-stabilised abundances of microbial populations with significant abundance changes over time, sorted by largest absolute log2 fold change, set 6 of 7. Significance is indicated with horizontal bars with accompanying log2 fold change (thresholds ||LFC|| > 1 and base mean > 50) and FDR-adjusted p-values indicated by asterisks (* < 0.05, ** < 0.01, *** 0.001).*

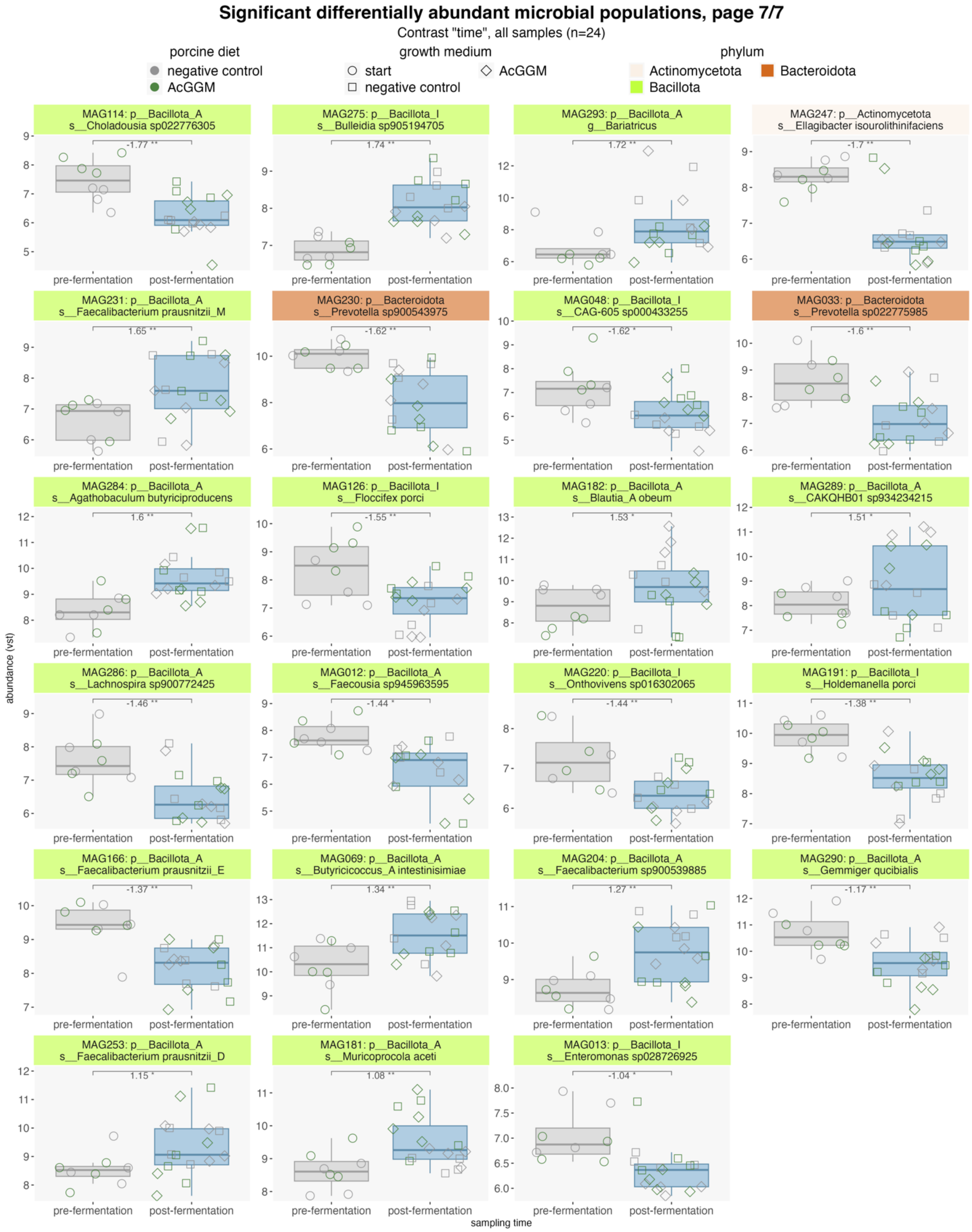

Figure SC1 continued. *Variance-stabilised abundances of microbial populations with significant abundance changes over time, sorted by largest absolute log2 fold change, set 7 of 7. Significance is indicated with horizontal bars with accompanying log2 fold change (thresholds ||LFC|| > 1 and base mean > 50) and FDR-adjusted p-values indicated by asterisks (* < 0.05, ** < 0.01, *** 0.001).*

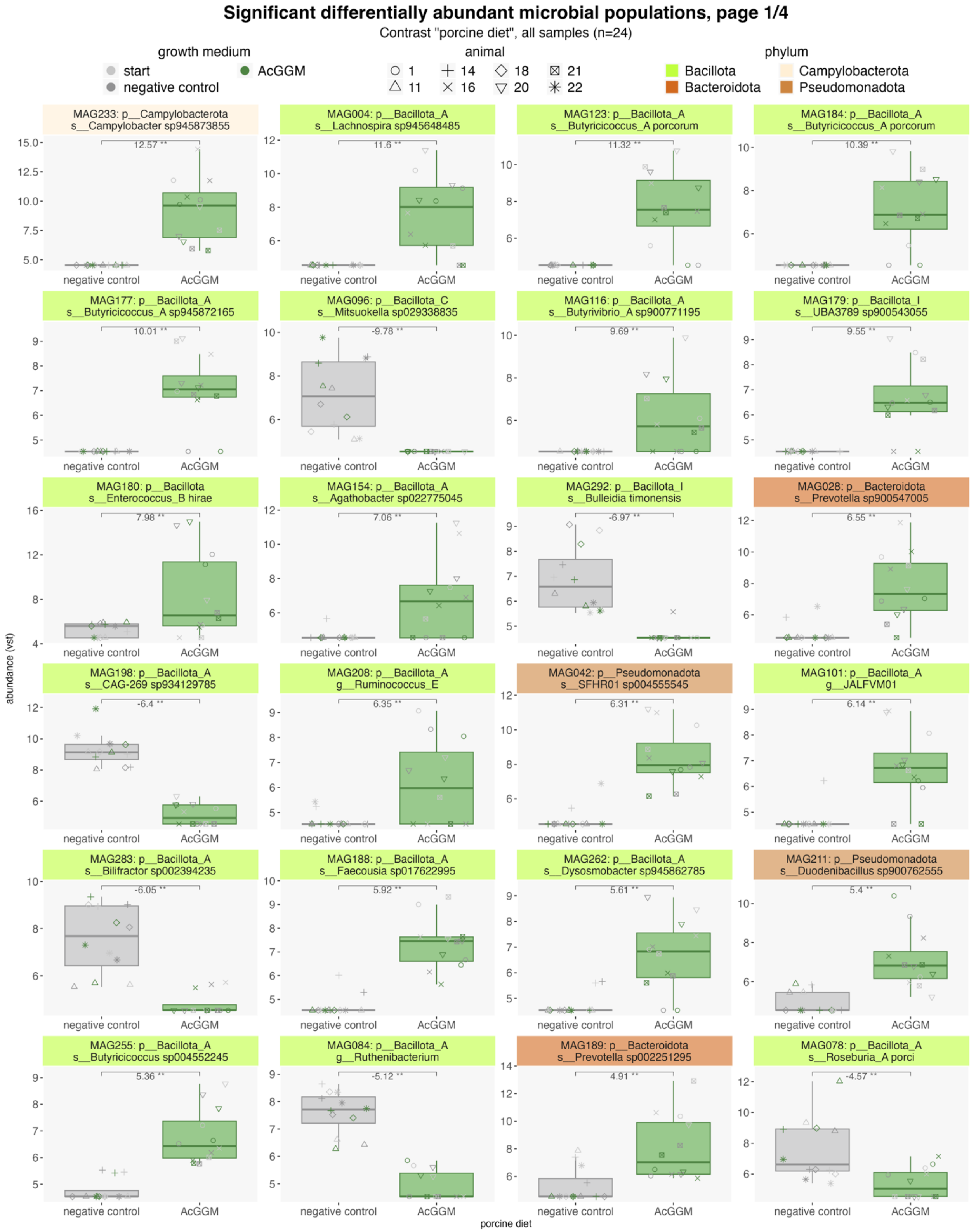

Figure SC2. *Variance-stabilised abundances of microbial populations with significant abundance differences across porcine diet groups, sorted by largest absolute log2 fold change, set 1 of 4. Significance is indicated with horizontal bars with accompanying log2 fold change (thresholds ||LFC|| > 1 and base mean > 50) and FDR-adjusted p-values indicated by asterisks (* < 0.05, ** < 0.01, *** 0.001).*

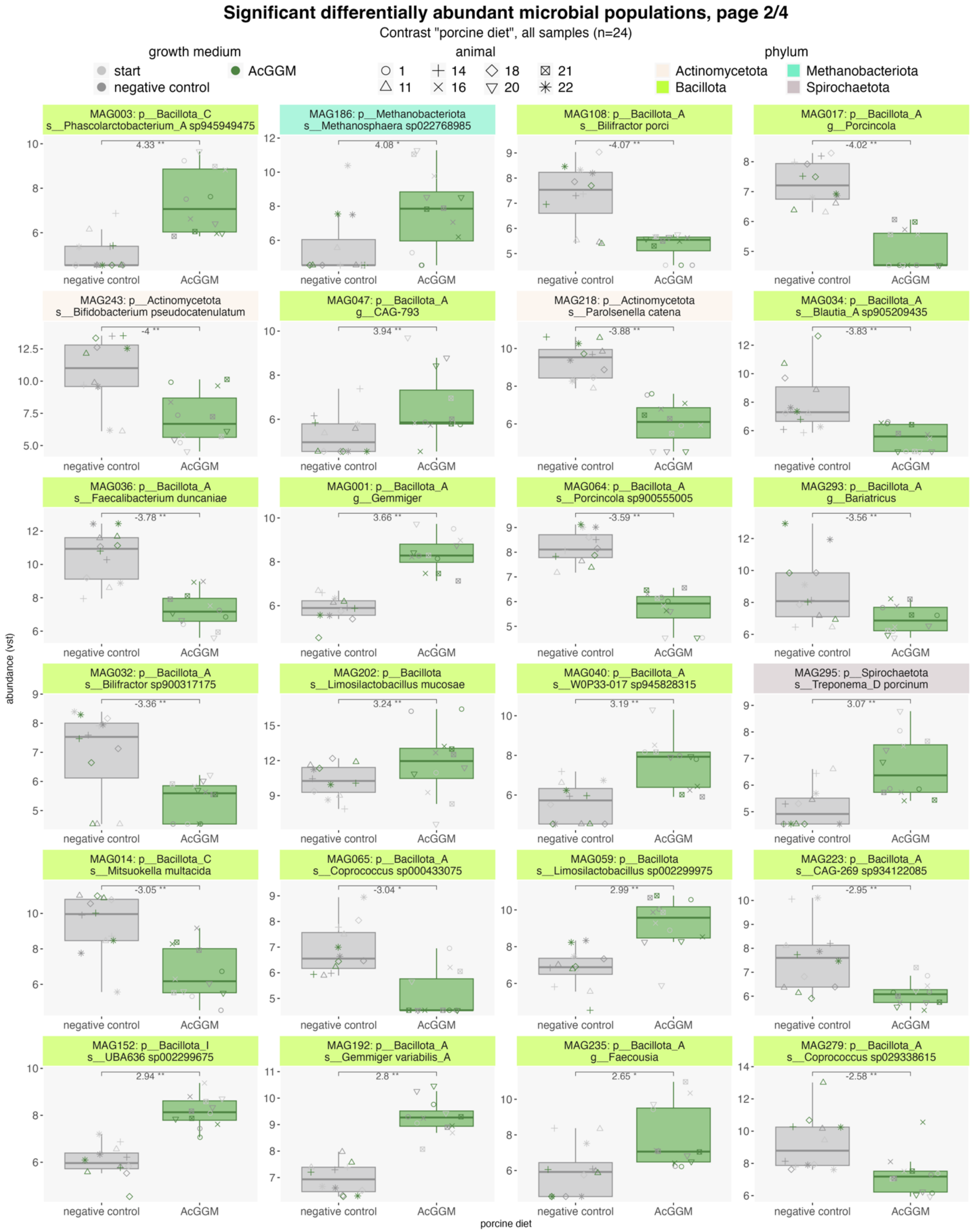

Figure SC2 continued. *Variance-stabilised abundances of microbial populations with significant abundance differences across porcine diet groups, sorted by largest absolute log2 fold change, set 2 of 4. Significance is indicated with horizontal bars with accompanying log2 fold change (thresholds ||LFC|| > 1 and base mean > 50) and FDR-adjusted p-values indicated by asterisks (* < 0.05, ** < 0.01, *** 0.001).*

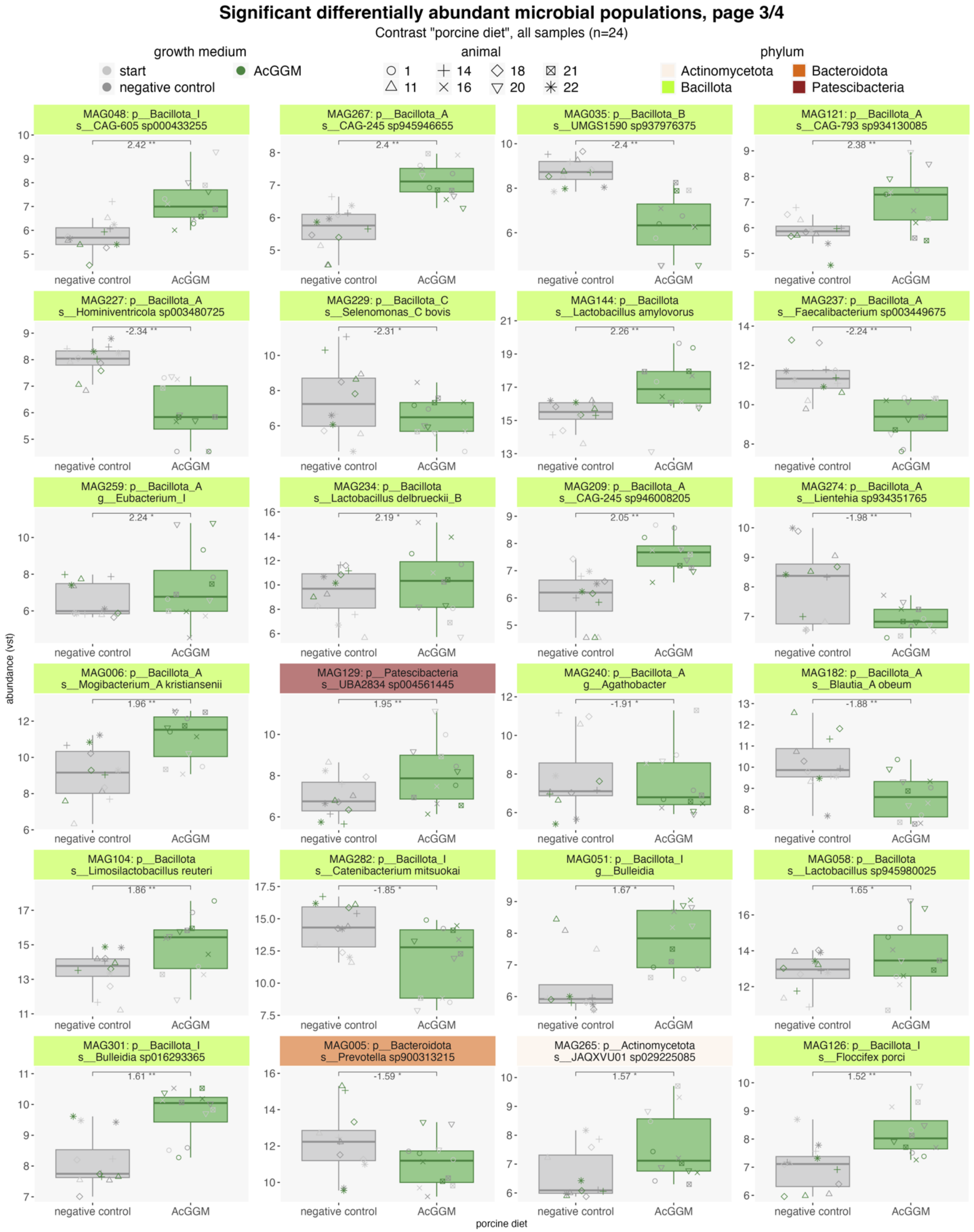

Figure SC2 continued. *Variance-stabilised abundances of microbial populations with significant abundance differences across porcine diet groups, sorted by largest absolute log2 fold change, set 3 of 4. Significance is indicated with horizontal bars with accompanying log2 fold change (thresholds ||LFC|| > 1 and base mean > 50) and FDR-adjusted p-values indicated by asterisks (* < 0.05, ** < 0.01, *** 0.001).*

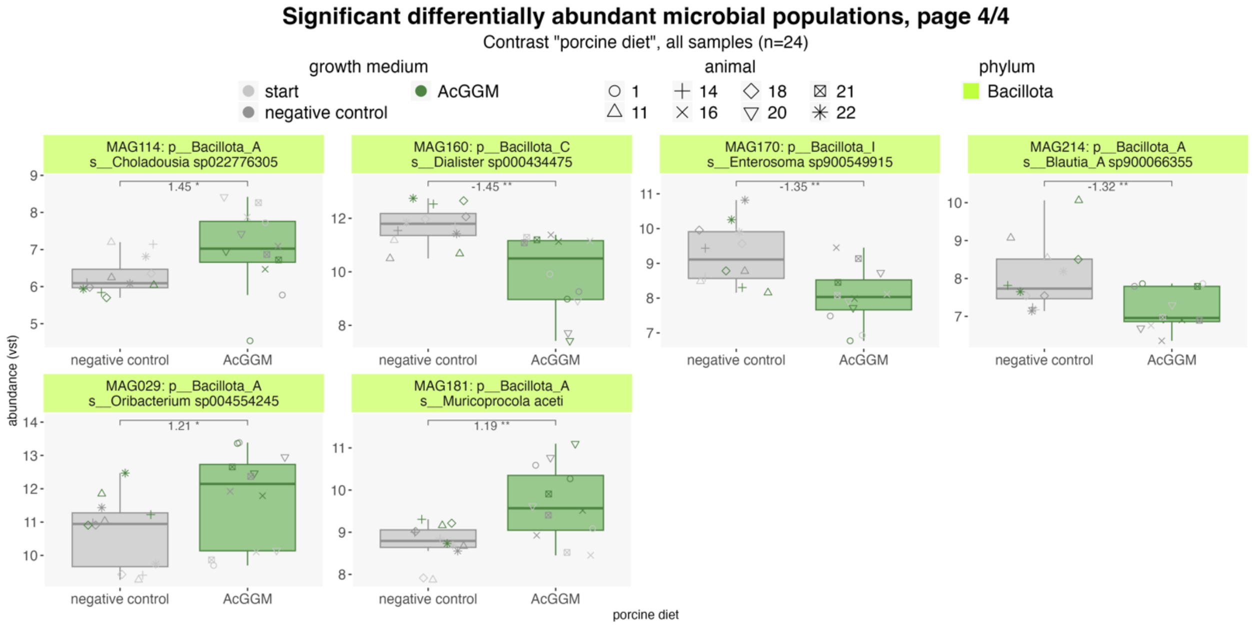

***Figure SC2 continued**. Variance-stabilised abundances of microbial populations with significant abundance differences across porcine diet groups, sorted by largest absolute log2 fold change, set 4 of 4. Significance is indicated with horizontal bars with accompanying log2 fold change (thresholds ||LFC|| > 1 and base mean > 50) and FDR-adjusted p-values indicated by asterisks (* < 0.05, ** < 0.01, *** 0.001).*

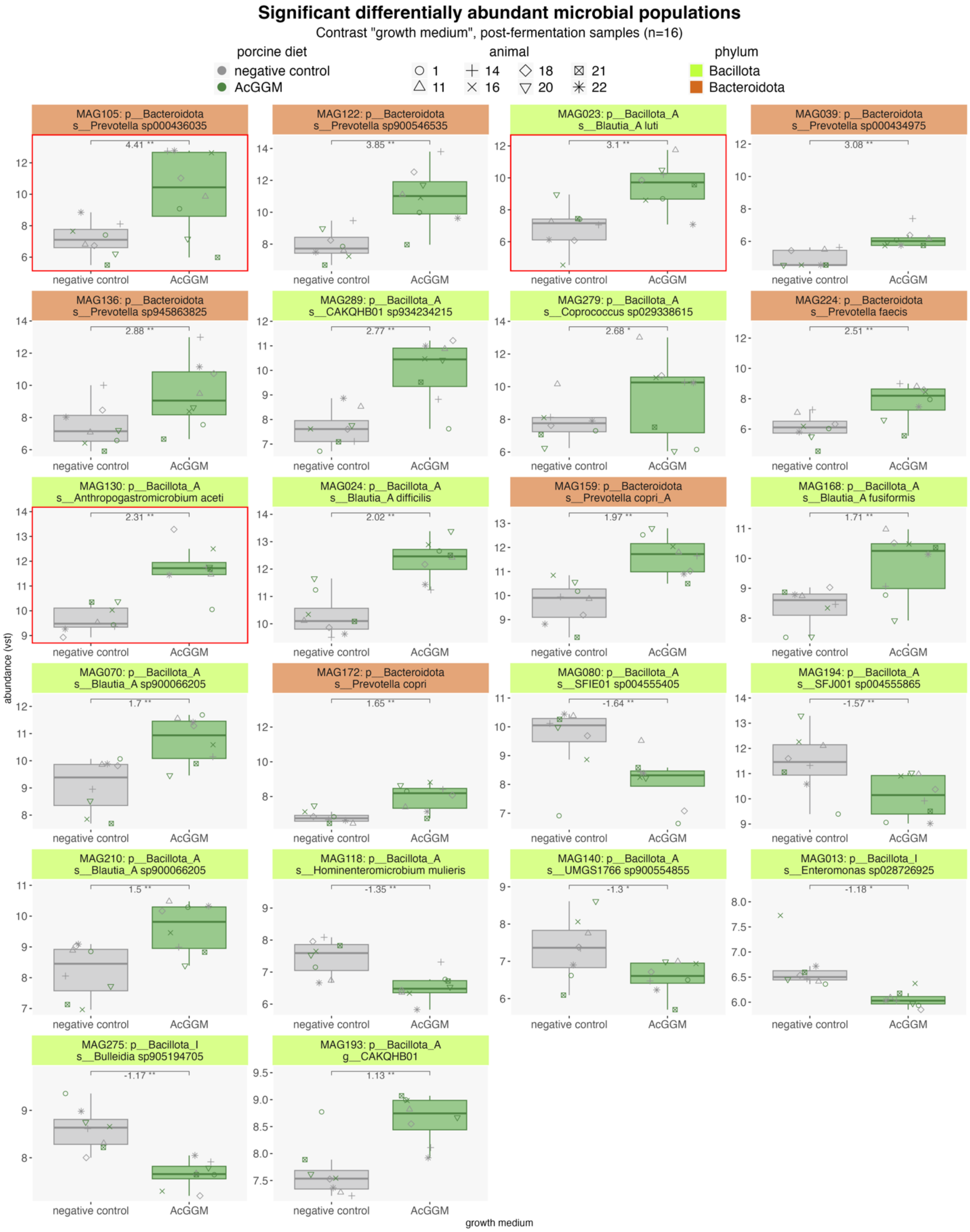

Figure SC3. *Variance-stabilised abundances of microbial populations with significant abundance differences across growth medium groups, sorted by largest absolute log2 fold change. Significance is indicated with horizontal bars with accompanying log2 fold change (thresholds ||LFC|| > 1 and base mean > 50) and FDR-adjusted p-values indicated by asterisks (* < 0.05, ** < 0.01, *** 0.001). Populations suggested to be new AcGGM degraders have been highlighted with a red frame.*

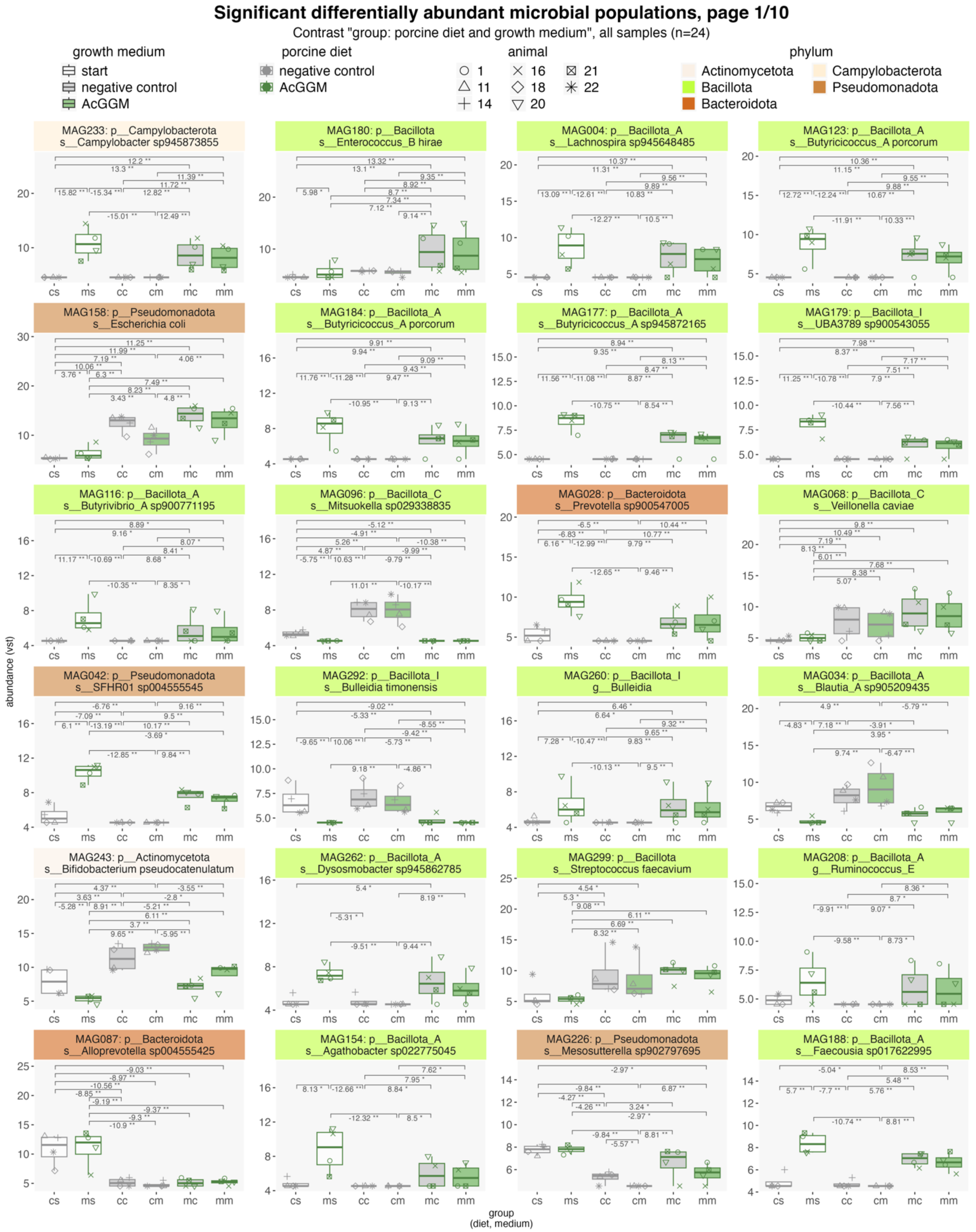

Figure SC4. *Variance-stabilised abundances of microbial populations with significant differences across any porcine diet and growth medium combination, sorted by largest absolute log2 fold change, set 1 of 10. Significance between groups is indicated with horizontal bars, log2 fold change (thresholds ||LFC|| > 1 and base mean > 50) and FDR-adjusted p-values indicated by asterisks (* < 0.05, ** < 0.01, *** 0.001).*

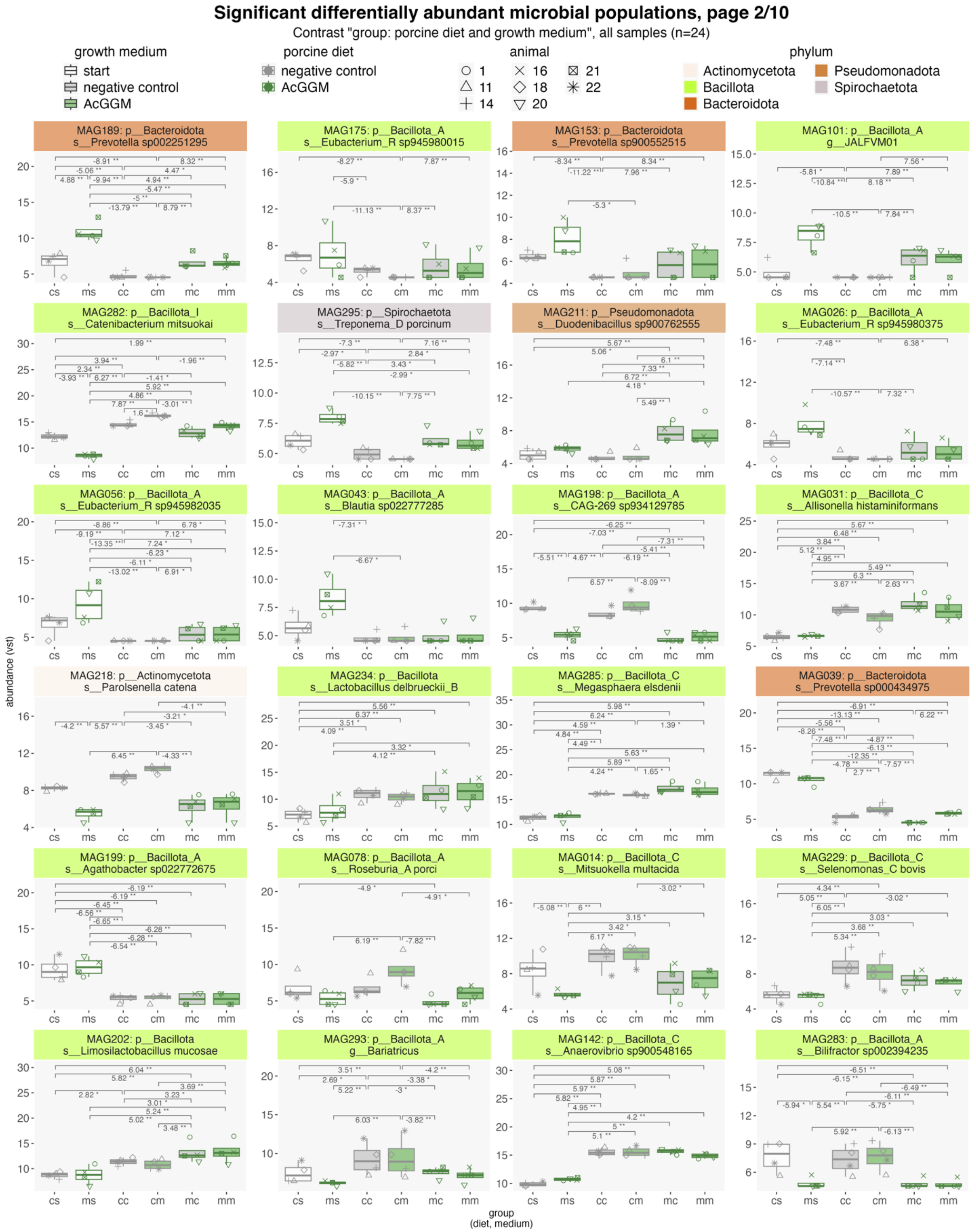

Figure SC4 continued. *Variance-stabilised abundances of populations with significant difference across any porcine diet and growth medium combination, sorted by largest absolute log2 fold change, set 2 of 10. Significance between groups is indicated with horizontal bars, log2 fold change (thresholds ||LFC|| > 1 and base mean > 50) and FDR-adjusted p-values indicated by asterisks (* < 0.05, ** < 0.01, *** 0.001).*

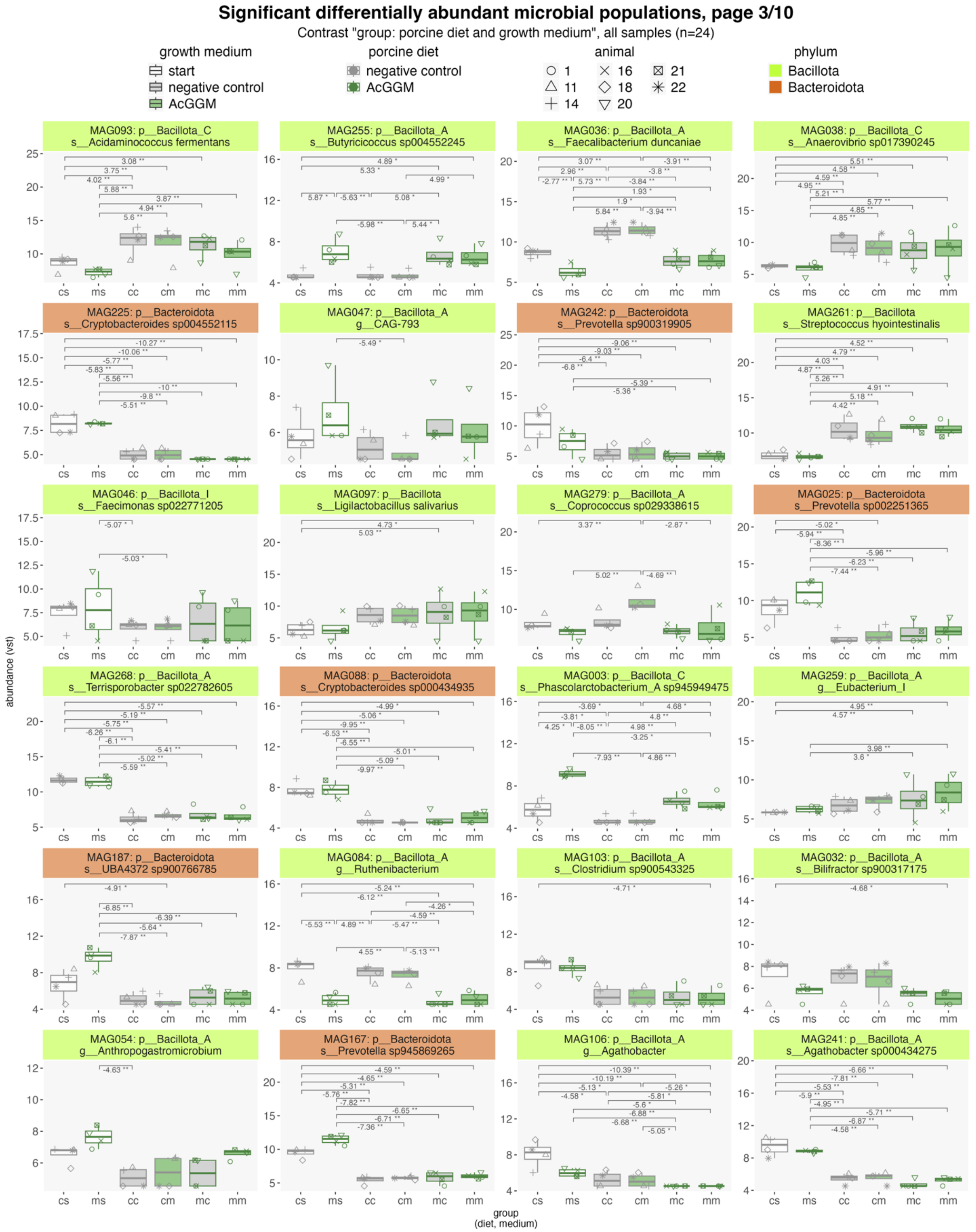

Figure SC4 continued. *Variance-stabilised abundances of populations with significant difference across any porcine diet and growth medium combination, sorted by largest absolute log2 fold change, set 3 of 10. Significance between groups is indicated with horizontal bars, log2 fold change (thresholds ||LFC|| > 1 and base mean > 50) and FDR-adjusted p-values indicated by asterisks (* < 0.05, ** < 0.01, *** 0.001).*

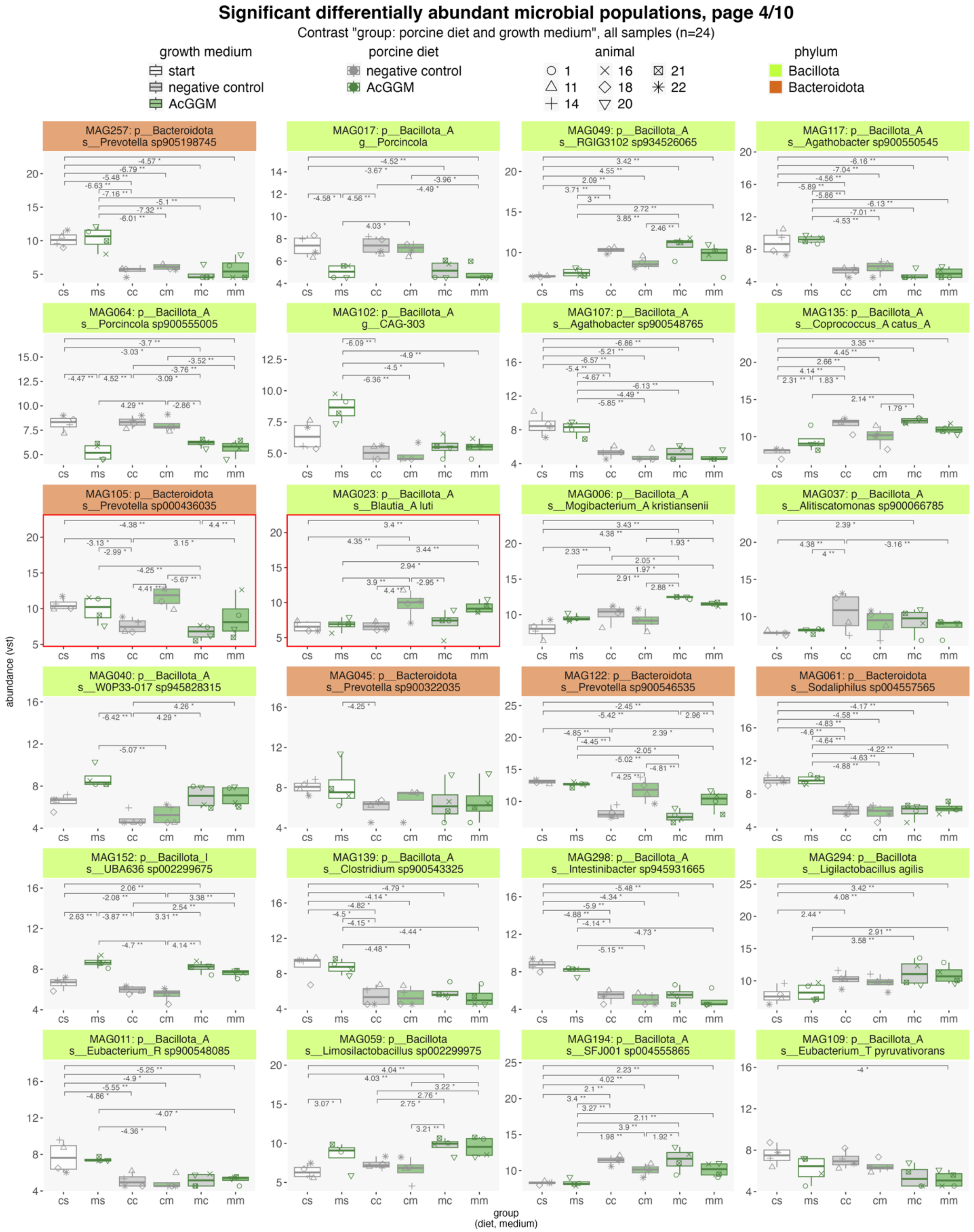

***Figure SC4 continued**. Variance-stabilised abundances of populations with significant difference across any porcine diet and growth medium combination, sorted by largest absolute log2 fold change, set 4 of 10. Significance between groups is indicated with horizontal bars, log2 fold change (thresholds ||LFC|| > 1 and base mean > 50) and FDR-adjusted p-values indicated by asterisks (* < 0.05, ** < 0.01, *** 0.001). Populations suggested to be new AcGGM degraders have been highlighted with a red frame.*

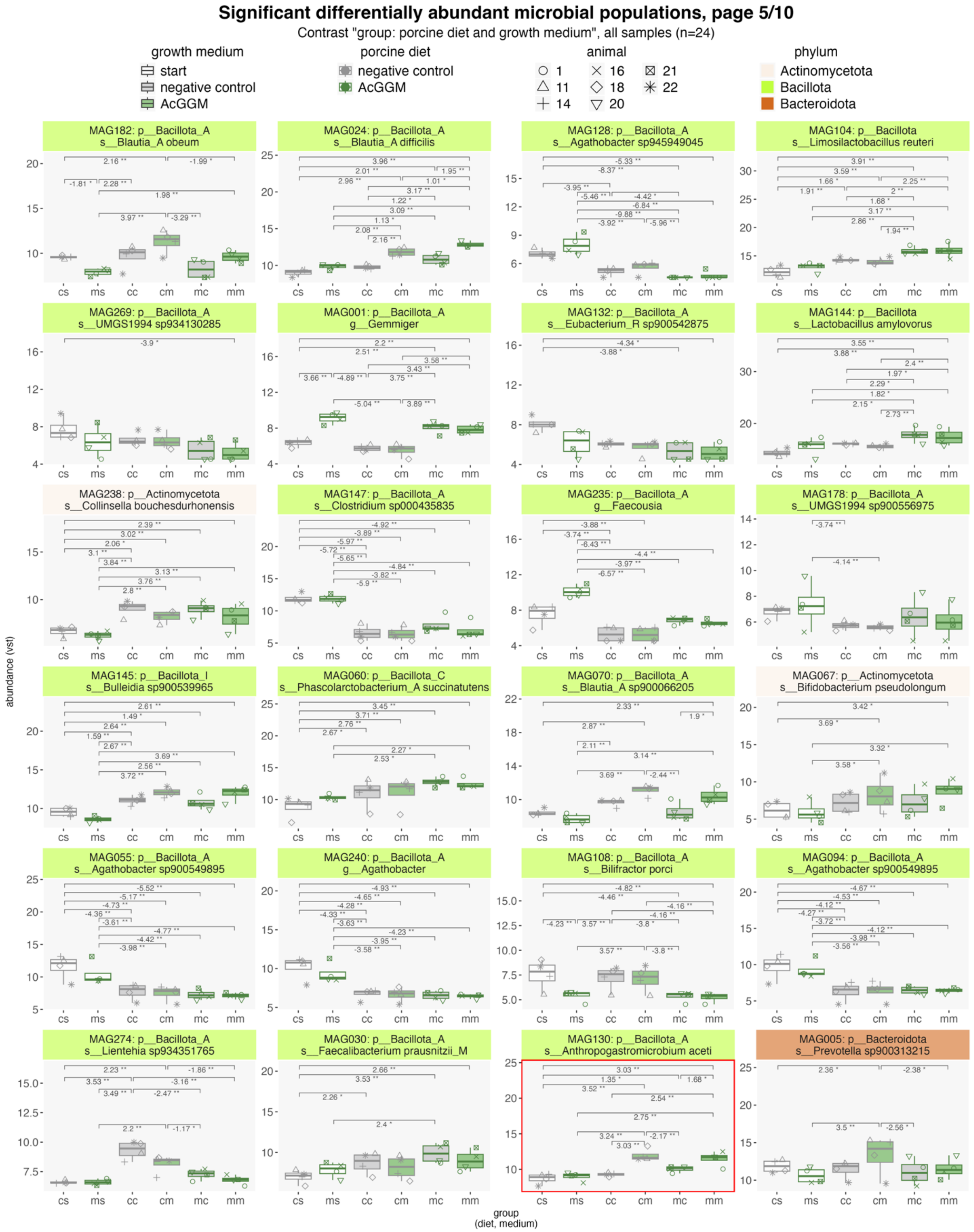

Figure SC4 continued. *Variance-stabilised abundances of populations with significant difference across any porcine diet and growth medium combination, sorted by largest absolute log2 fold change, set 5 of 10. Significance between groups is indicated with horizontal bars, log2 fold change (thresholds ||LFC|| > 1 and base mean > 50) and FDR-adjusted p-values indicated by asterisks (* < 0.05, ** < 0.01, *** 0.001). Populations suggested to be new AcGGM degraders have been highlighted with a red frame.*

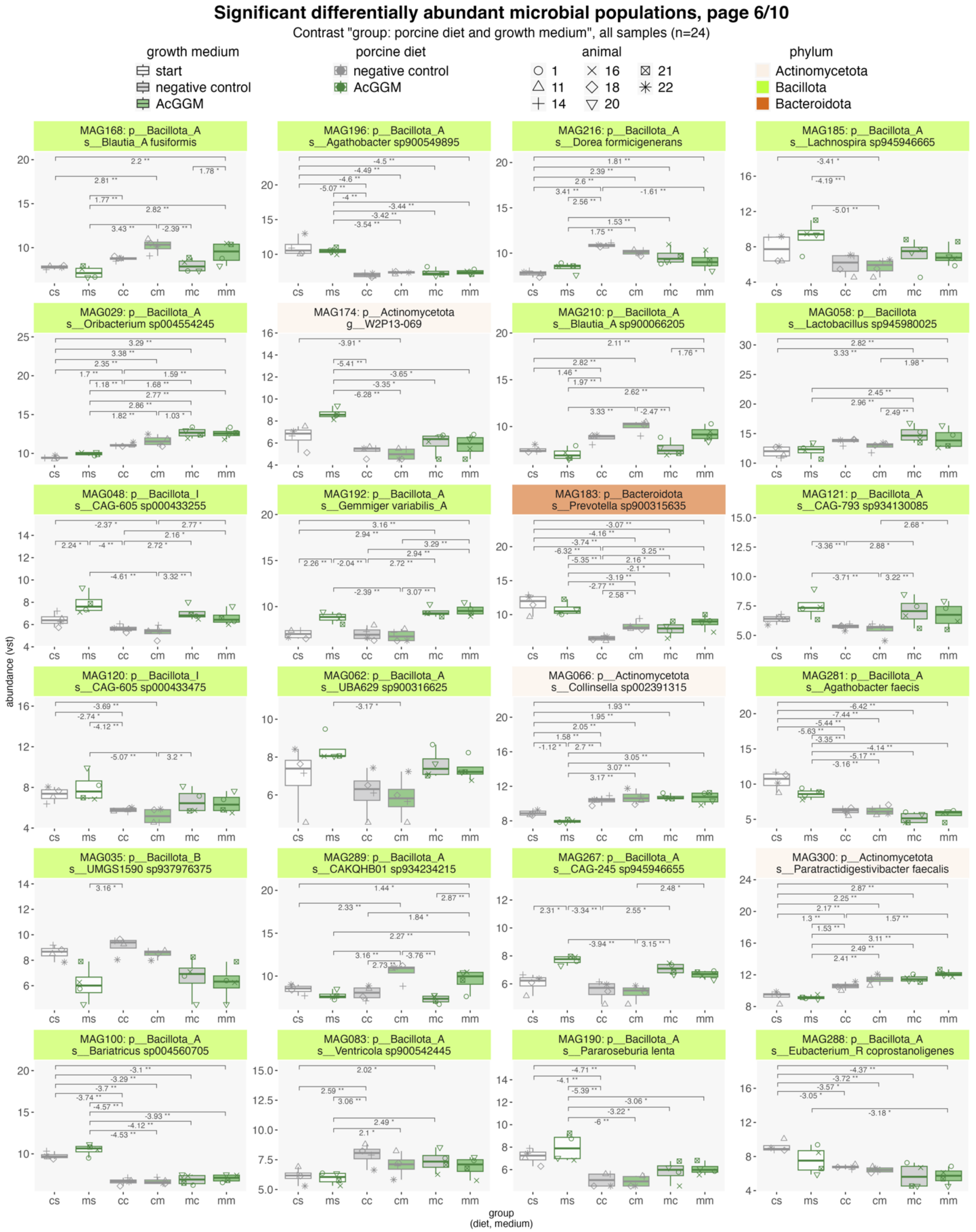

Figure SC4 continued*. Variance-stabilised abundances of populations with significant difference across any porcine diet and growth medium combination, sorted by largest absolute log2 fold change, set 6 of 10. Significance between groups is indicated with horizontal bars, log2 fold change (thresholds ||LFC|| > 1 and base mean > 50) and FDR-adjusted p-values indicated by asterisks (* < 0.05, ** < 0.01, *** 0.001).*

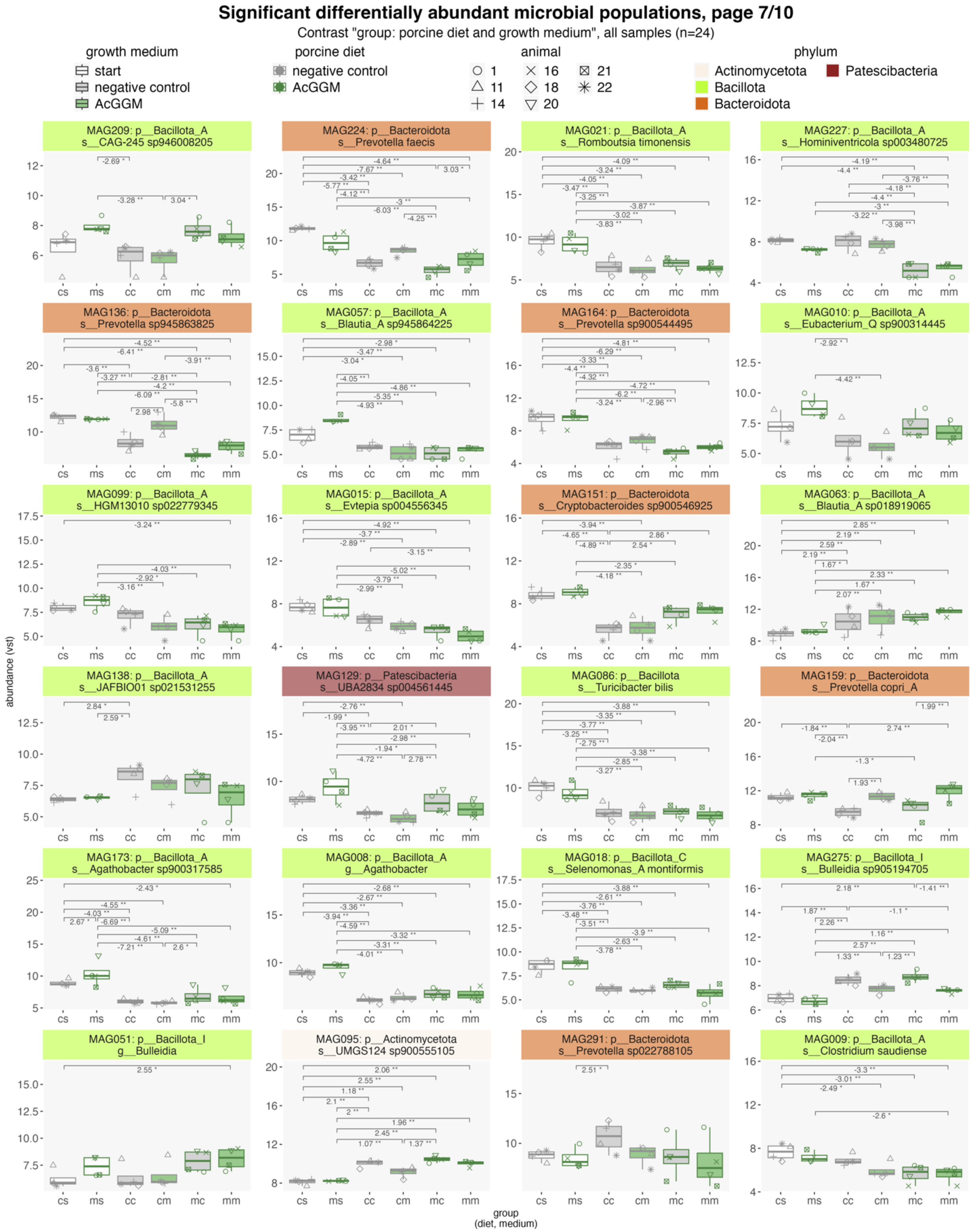

Figure SC4 continued. *Variance-stabilised abundances of populations with significant difference across any porcine diet and growth medium combination, sorted by largest absolute log2 fold change, set 7 of 10. Significance between groups is indicated with horizontal bars, log2 fold change (thresholds ||LFC|| > 1 and base mean > 50) and FDR-adjusted p-values indicated by asterisks (* < 0.05, ** < 0.01, *** 0.001).*

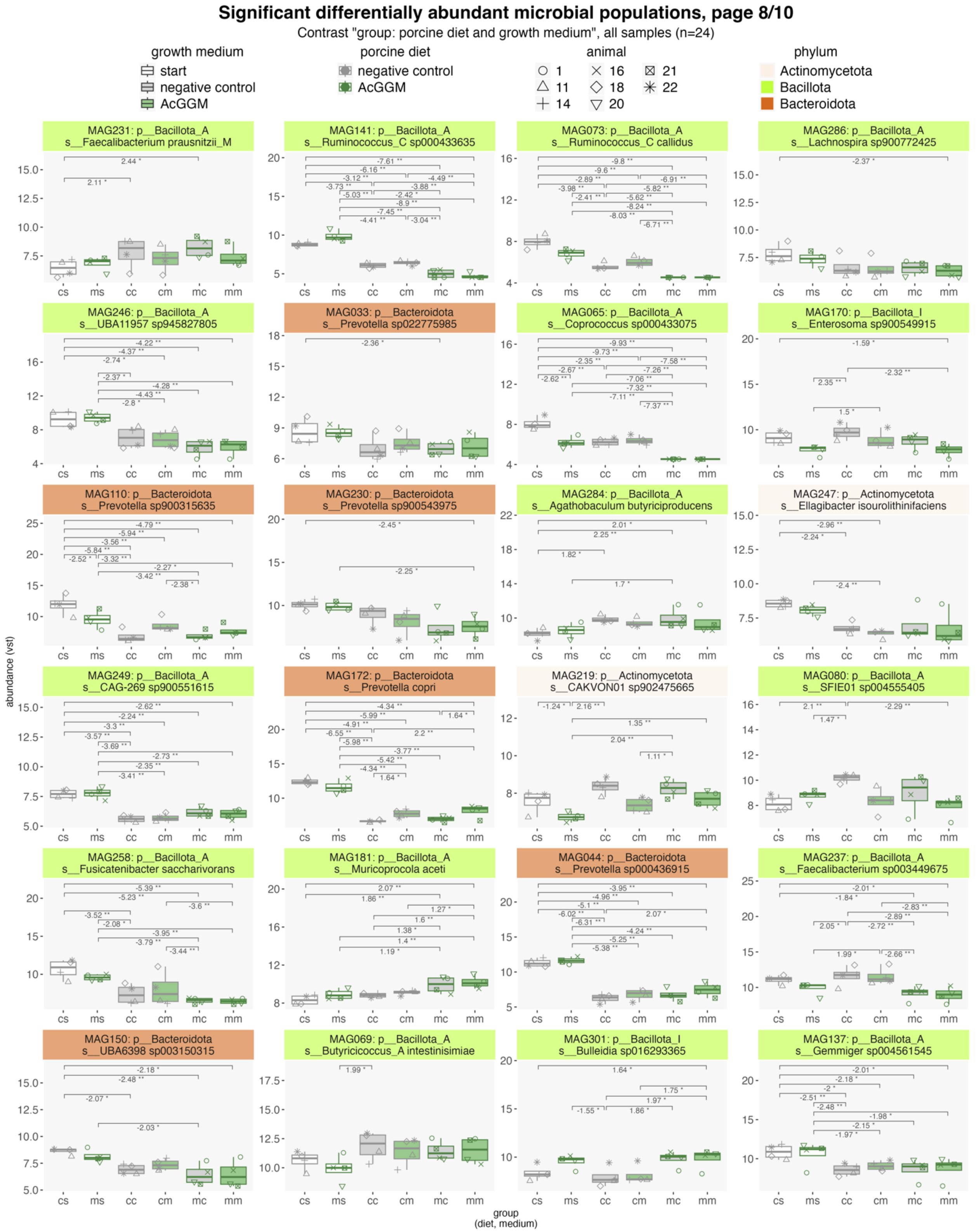

Figure SC4 continued. *Variance-stabilised abundances of populations with significant difference across any porcine diet and growth medium combination, sorted by largest absolute log2 fold change, set 8 of 10. Significance between groups is indicated with horizontal bars, log2 fold change (thresholds ||LFC|| > 1 and base mean > 50) and FDR-adjusted p-values indicated by asterisks (* < 0.05, ** < 0.01, *** 0.001).*

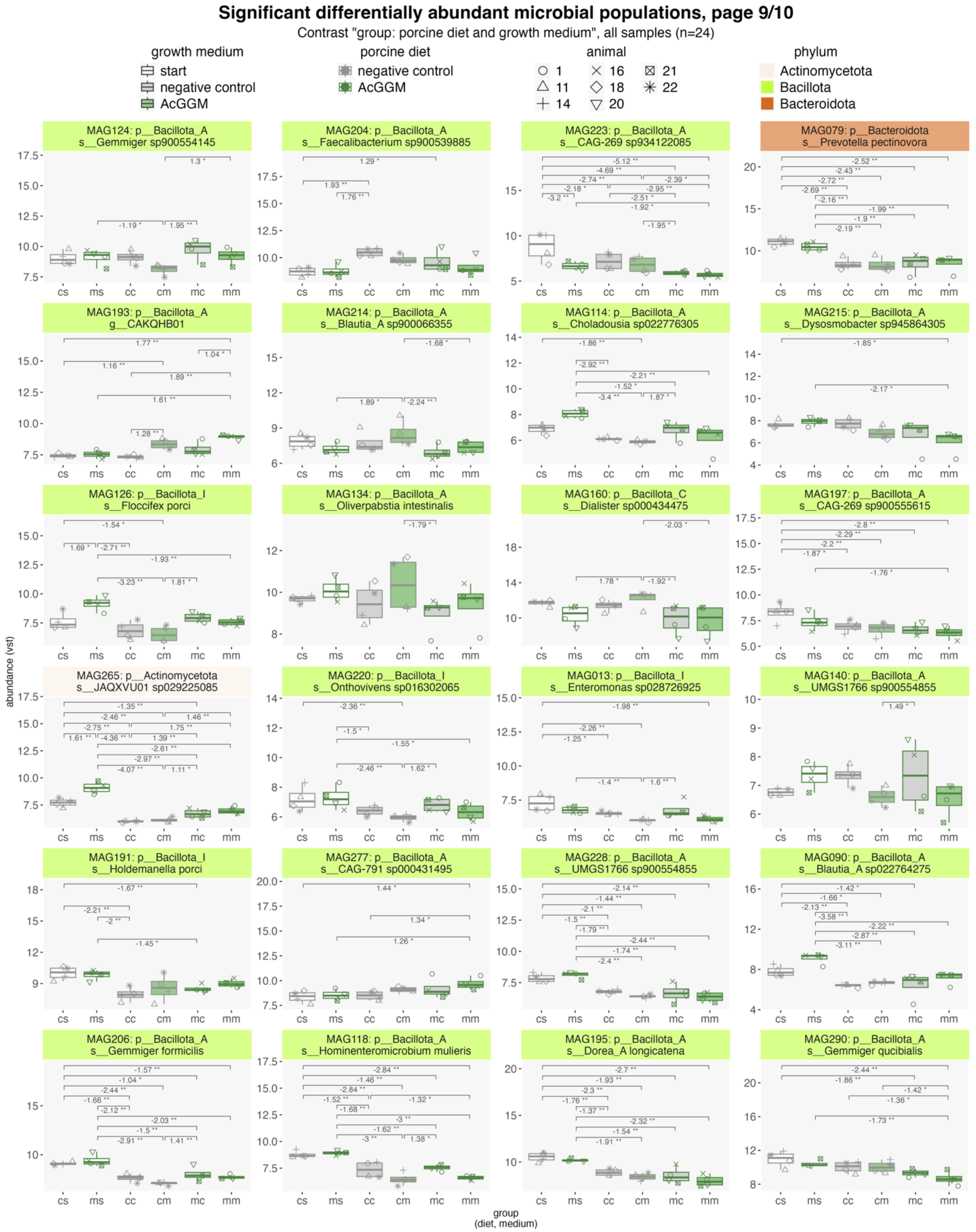

Figure SC4 continued. *Variance-stabilised abundances of populations with significant difference across any porcine diet and growth medium combination, sorted by largest absolute log2 fold change, set 9 of 10. Significance between groups is indicated with horizontal bars, log2 fold change (thresholds ||LFC|| > 1 and base mean > 50) and FDR-adjusted p-values indicated by asterisks (* < 0.05, ** < 0.01, *** 0.001).*

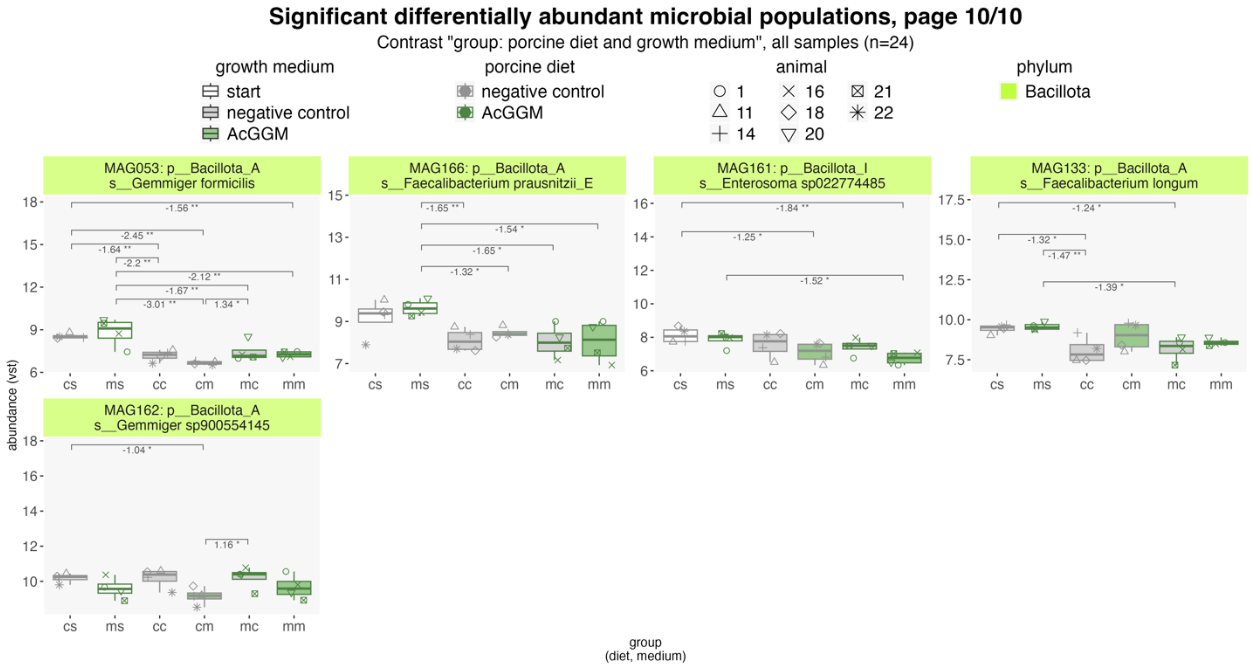

Figure SC4 continued. *Variance-stabilised abundances of populations with significant difference across any porcine diet and growth medium combination, sorted by largest absolute log2 fold change, set 10 of 10. Significance between groups is indicated with horizontal bars, log2 fold change (thresholds ||LFC|| > 1 and base mean > 50) and FDR-adjusted p-values indicated by asterisks (* < 0.05, ** < 0.01, *** 0.001).*
