# Supplementary material for: In vitro model reveals structural and metabolic insights into the porcine cecal microbiota in response to β-mannan exposure
Source: Appl Environ Microbiol. 2026 Jun 18;92(7):e00140-26. doi: 10.1128/aem.00140-26 (PMC13390407; doi:10.1128/aem.00140-26)
Supplement: Supplemental File D — Short-chain fatty acid data. [file aem.00140-26-s0004.pdf]

In vitro model reveals structural and metabolic insights to the porcine caecal microbiota in response to β-mannan exposure

This supplementary contains additional visualisations of the concentrations of short-chained fatty acids measured in samples following *in vitro* fermentation (**Fig. SD1**), and Pearson correlation between these concentrations and metagenome-assembled genome abundances (**Fig. SD2**).

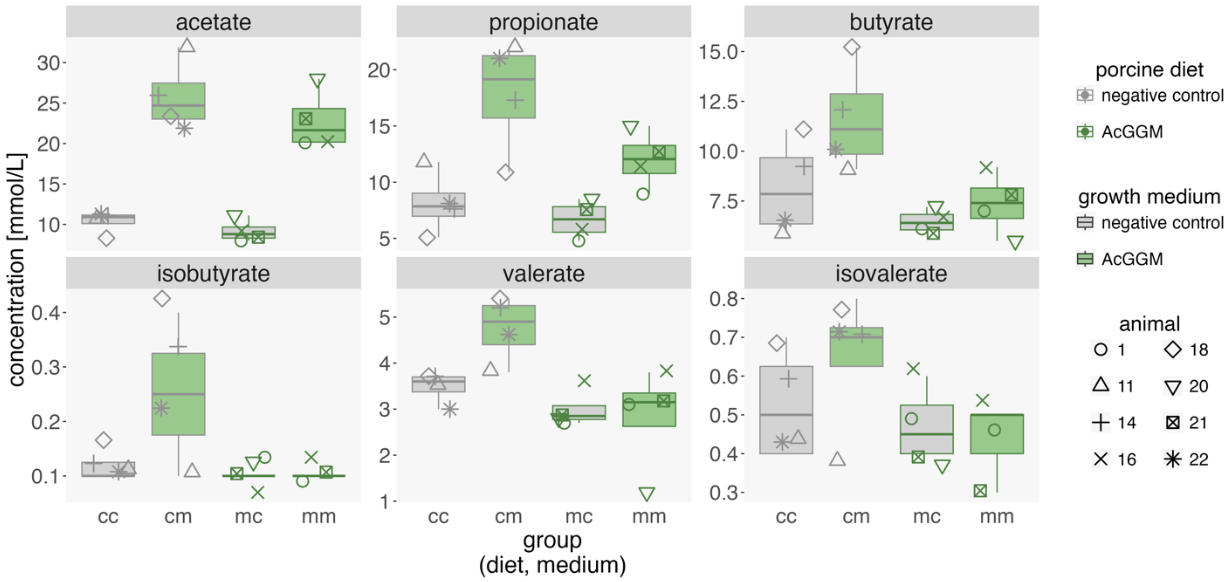

***Figure SD1**. Concentrations of the measured short chain fatty acids across sample groups of different porcine diets and in vitro growth media.*

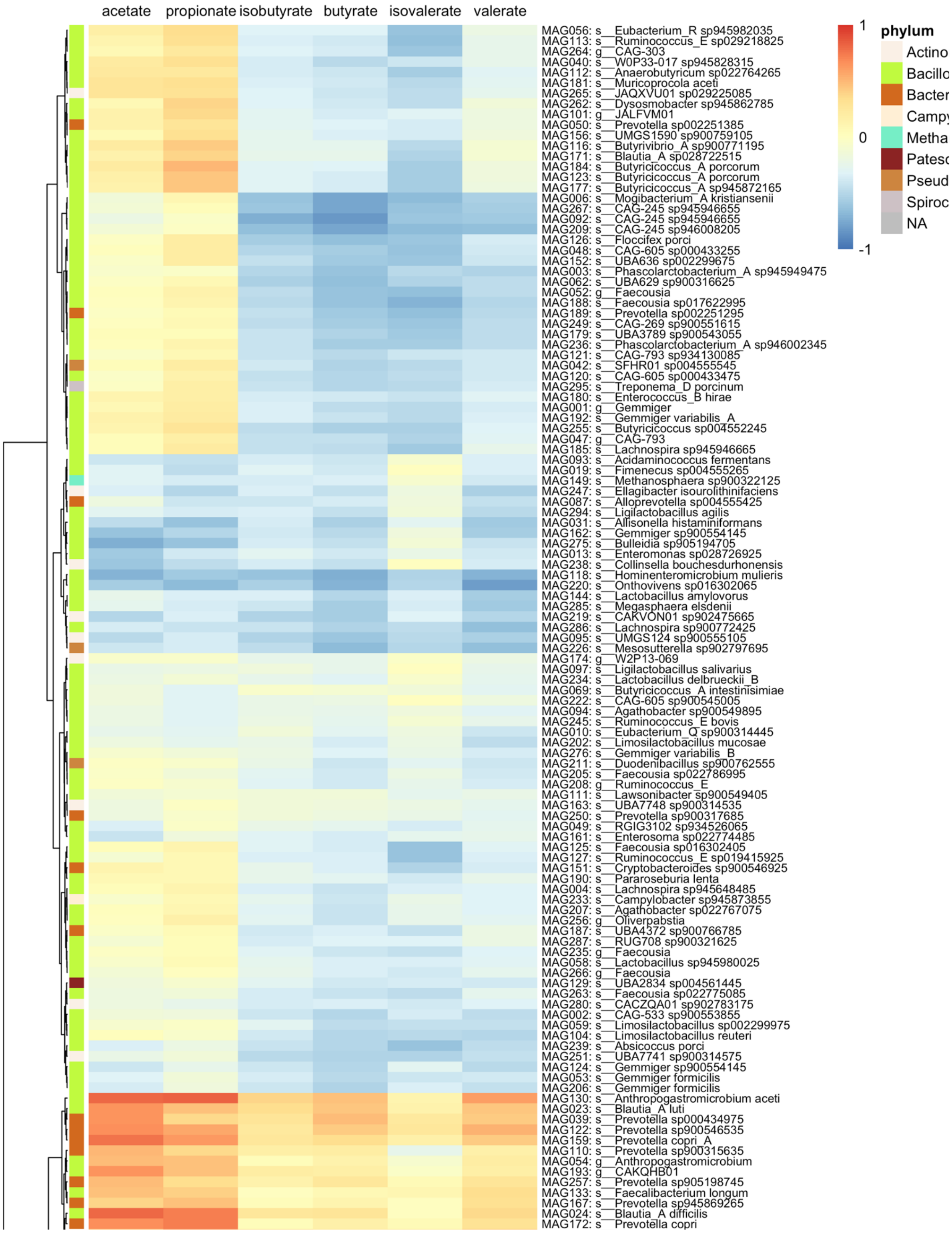

Figure SD2*. Pearson correlation of short-chained fatty acid concentrations with population abundances, set 1 of 3. Correlation values range from blue (-1) to red (1). Rows are clustered using Euclidean distance and Ward D2 criterion, and annotated with the phylum of each respective population.*

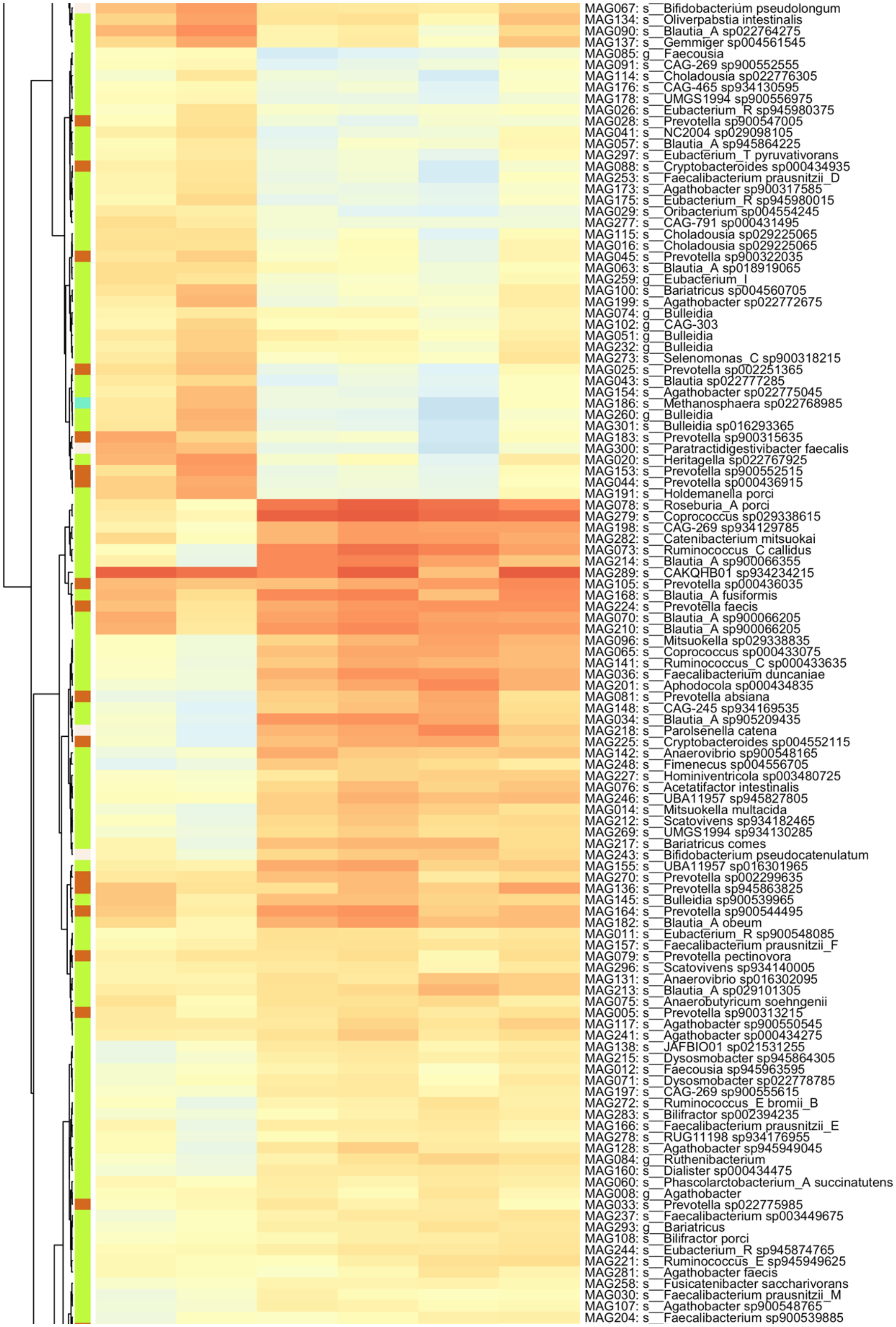

***Figure SD2 continued**. Pearson correlation of short-chained fatty acid concentrations with population abundances, set 2 of 3. Correlation values range from blue (-1) to red (1). Rows are clustered using Euclidean distance and Ward D2 criterion, and annotated with the phylum of each respective population.*

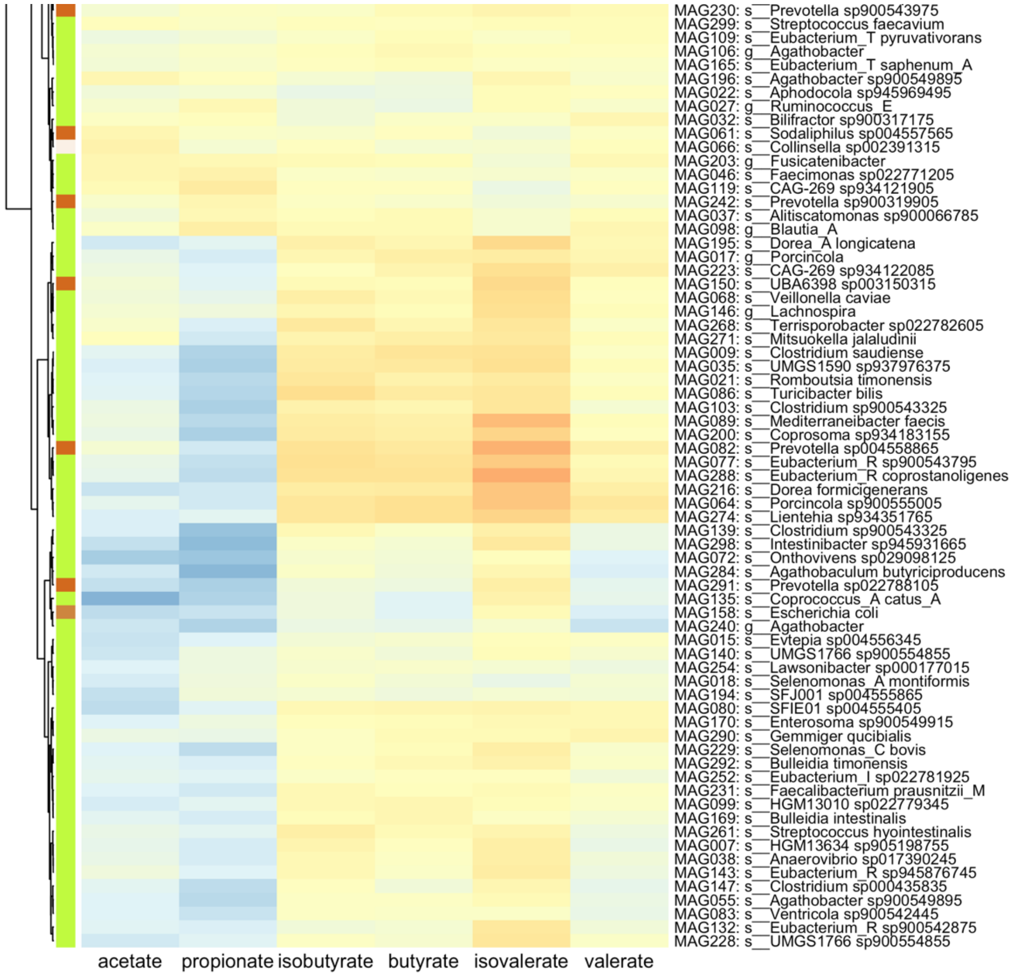

Figure SD2 continued. *Pearson correlation of short-chained fatty acid concentrations with population abundances, set 3 of 3. Correlation values range from blue (-1) to red (1). Rows are clustered using Euclidean distance and Ward D2 criterion, and annotated with the phylum of each respective population.*
